# Lactoferrins in Their Interactions with Molecular Targets: A Structure-Based Overview

**DOI:** 10.3390/ph17030398

**Published:** 2024-03-20

**Authors:** Roberta Piacentini, Alberto Boffi, Edoardo Milanetti

**Affiliations:** 1 Department of Physics, University “Sapienza”, Piazzale Aldo Moro 5, 00185 Rome, Italy; edoardo.milanetti@uniroma1.it; 2 Department of Biochemical Sciences, University “Sapienza”, Piazzale Aldo Moro 5, 00185 Rome, Italy; alberto.boffi@uniroma1.it; 3 Center for Life Nano and Neuro Science, Istituto Italiano di Tecnologia (IIT), Viale Regine Elena 291, 00161 Rome, Italy

**Keywords:** lactoferrin, antimicrobial activity, antiviral activity, lactoferrin-derived peptides, mechanism of action

## Abstract

Lactoferrins and lactoferrin-derived peptides display numerous functions linked to innate immunity in mammalians, spanning from antimicrobial to anti-inflammatory and immunomodulatory actions, and even demonstrate antitumor properties. To date, the proposed mechanisms for their biological actions are varied, although the molecular basis that governs lactoferrin interactions with molecular targets has been clarified only in a limited number of specific cases. However, key in silico methods have recently moved the topic to the fore, thus greatly expanding the possibilities of large-scale investigations on macromolecular interactions involving lactoferrins and their molecular targets. This review aims to summarize the current knowledge on the structural determinants that drive lactoferrin recognition of molecular targets, with primary focus on the mechanisms of activity against bacteria and viruses. The understanding of the structural details of lactoferrins’ interaction with their molecular partners is in fact a crucial goal for the development of novel pharmaceutical products.

## 1. Introduction

Lactoferrins belong to the “transferrin superfamily”, a group of well-conserved single-chain glycosylated proteins that transport iron from plasma to cells and contribute to the regulation of iron transport in biological fluids [1]. Most members of the superfamily display a similar fold and consist of two homologous lobes (N- and C-lobe) connected by a short hinge region [2]. Each of the two lobes is able to reversibly bind a single ferric ion. It is of interest to comment on the evolutionary path that underlines this superfamily, as follows. Early metazoans have been proposed to develop single-lobe iron-binding proteins able to uptake iron from sea water. Subsequent gene duplication and fusion led to the evolution of the primordial single-lobe iron-binding protein into the bi-lobed mammalian transferrins (Tf) and lactoferrins (Lf) [3,4]. In this model, Lambert and colleagues suggested that Lf originated from a subsequent gene duplication event within the mammalian lineage that occured approximately 125 million years ago [1,3]. In mammalians, lactoferrins are expressed by epithelial cells in diverse tissues in mammals and are found in virtually all bodily exocrine secretions comprising colostrum and milk, tears, nasal and bronchial secretions, and saliva [5]. Furthermore, lactoferrins are synthesized by the hematopoietic tissue in the bone marrow, and they are present in the granules of polymorphonuclear neutrophils [6]. Other exocrine secretions contain lactoferrins at reduced levels [5].

**Figure 1:**
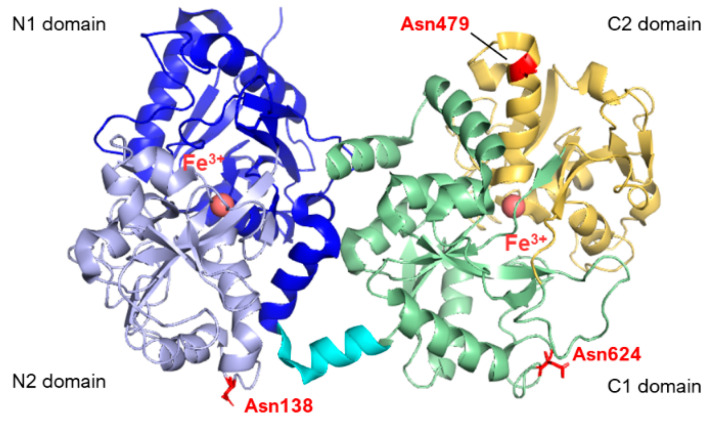
Structure of human lactoferrin. Lobes N and C are represented in different colors (blue and lavender for N1 and N2, green and yellow for C1 and C2, respectively), and the α-helix connecting the two domains is in cyan. The three glycosylation sites are highlighted in red (PDB: 2BJJ). Image created with PyMOL Molecular Graphic System version 3.0 Schrödinger, LLC.

From the structural point of view, lactoferrins are monomeric glycoproteins of about 690 amino acids (average molecular mass of ~80 kDa), and their three-dimensional structures have been reported for five species comprising human [7,8,9], cow [2,10], camel [11], buffalo [12,13], and horse [14], all of them sharing very high structural similarity. The single polypeptide chain of lactoferrins is folded into two globular lobes sharing ~40% sequence homology and referred to as the N-lobe (amino acids 1–333) and C-lobe (amino acids 345–691, in humans). Each lobe can be further divided into two domains, namely the N1 domain (amino acids 1–90, 251–333) and the N2 domain (amino acids 91–250), the C1 domain (amino acids 345–431, 593–689), and the C2 domain (amino acids 432–592). The two lobes are connected by a 10 amino acid-long (334 to 344 in humans) three-turn α-helix segment (see Figure 1). Each pair of N1 and N2 and C1 and C2 domains harbor an iron-binding site. Four amino acids (two Tyr, Asp, His) are the main ligands of Fe^3+^ in each lobe. In addition, two oxygens from the synergistic carbonate anion complete the architecture of the binding site, accompanied by a portion of the N-terminus and an arginine side chain that contribute to the electrostatic coordination of the carbonate itself.

Each lactoferrin molecule can, in principle, reversibly bind two iron ions, although the C-lobe site has a much higher intrinsic affinity for free ferric ions under physiological conditions (estimated K_D_ ≈ 10^−22^ M) due to very slow kinetics of metal release [15,16,17]. Conformational changes have been described in lactoferrins upon iron binding, indicating that iron-saturated species appear to adopt a more closed structure with respect to the iron-free species [8,16].

Lactoferrins exhibit heterogeneous glycosylation, meaning the number of sugar attachment sites varies across different species. Furthermore, even within the same species, this variability in glycosylation sites can depend on the specific tissue where the protein is expressed [18,19].

Glycosylation significantly influences lactoferrin’s stability and its resistance to being broken down by proteolytic enzymes, primarily by enhancing the solubility of proteins once they are secreted and by improving the ability of Lf to attach to certain types of cells or specific receptors. Nonetheless, this process has minimal impact on lactoferrin’s thermal stability or its capacity to bind to and release iron.

To date, human lactoferrin (hLf) has three potential N-glycosylation sites comprising Asn138, Asn479, and Asn624, although the site at 479 is found to be glycosylated only in 5% of the mature proteins in body fluids [20,21]. In contrast, bovine lactoferrin displays five potential glycosylation sites, namely Asn233, Asn281, Asn368, Asn476, and Asn545, whose populations among the molecules within milk also show heterogeneity [22,23]. The N-linked oligosaccharides belong to the class of high-mannose glycans, characterized by a N-acetylglucosamine (GlcNAc) core modified with mannose (Man) residues. By contrast, in the case of complex N-glycans, the common core pentasaccharide, consisting of Man3GlcNAc2, is elongated with GlcNAc at the α1,3- and α1,6-linked mannose residues. Additional monosaccharides, such as galactose, fucose and sialic acids, can also be added to complex N-glycans. The oligosaccharide moieties of Lf have demonstrated modulatory effects on the biological functions of lactoferrin beyond their pivotal intracellular role in protein folding, oligomerization, quality control, sorting, and transport. N-glycans, in particular, have been shown to enhance iron binding [24,25] and affect the anti-adhesive capacities of Lf towards bacterial or viral species [26]. The presence of sialic acid moieties within the oligosaccharide skeleton also appears to play a significant role in molecular recognition and protein stability [27].

As a whole, the body of high-resolution structural data currently available is providing the first clues on the molecular targets of Lfs that could explain some of the key biological activities reported to date. In particular, antibacterial activity of lactoferrins and its peptides has been elucidated in structural terms for those antimicrobial activities of lactoferrin that are related to iron chelation. In this framework, lactoferrins also impact immune homeostasis and modulate inflammatory responses, which is likely to contribute to the host-parasite interaction. Again, these activities could be further discussed in the framework of iron-binding ability through the modulation of oxidative stress caused by reactive oxygen species within the inflammatory response or by modulation effect on the innate and adaptive immune responses by maturation and differentiation of immune cells.

Novel potential Lfs activities have been demonstrated recently in connection with reported anticancer properties. In this case, several targets have been proposed, on the basis of in silico docking studies, that are linked to cancer cell growth: induction of apoptosis [28], and inhibition of cancer cell migration and invasion thus leading to confinement of metastasis [29].

### Further Insight on the C-Lobe of Lactoferrin

As discussed in the following sections of the present manuscript, the C-lobe of lactoferrin plays a crucial role in its antiviral and antibacterial actions. This binding is largely attributed to the interaction of positively charged regions, of the C-lobe in particular, with negatively charged components of the pathogens. In the case of viruses, lactoferrin’s C-lobe can bind to viral envelope glycoproteins.

For example, the C-lobe of bLf interacts with influenza A virus and prevents infection of different viral subtypes. Furthermore, it has been found that it also operates a protective role in preventing nonsteroidal anti-inflammatory drug (NSAID)-induced gastropathy [30]. Additionally, the C-lobe of lactoferrin has been shown to be involved in the anti-adenovirus activity as the N-lobe was able to inhibit adenovirus infection while the C-lobe was ineffective [31]. Moreover, the C-lobe of lactoferrin has been demonstrated to interfere with the fusogenic function of viral hemagglutinin, preventing influenza A virus infection [32].

The antibacterial activity of the C-lobe of lactoferrin is also noteworthy. It has been suggested that the antibacterial activity of the recombinant bovine lactoferrin C-lobe is related to its iron-binding activity or the presence of antibacterial peptides [33]. Furthermore, compared with the N-lobe, the C-lobe of transferrin has shown stronger growth inhibitory activity against *Escherichia coli*, indicating its potential antibacterial properties [34]. Additionally, synthetic peptides containing the C-lobe sequence of bovine lactoferrin have exhibited inhibitory effects on bacterial growth, highlighting the antibacterial potential of the C-lobe [35].

Finally, a more recent study by Jin et al. [36] provides valuable insights into the antibacterial activity of the C-lobe of bovine lactoferrin. The research highlights that lactoferrin, as an iron-binding protein, restricts the availability of free iron, which is essential for the growth of microorganisms. This limitation of free iron may contribute to the antibacterial activity of the recombinant bLf C-lobe. This finding aligns with previous research that suggests the antibacterial potential of the C-lobe of lactoferrin. Therefore, the ability of the C-lobe to bind Fe^3+^ is a crucial mechanism underlying its antibacterial action. By sequestering iron, the C-lobe of lactoferrin hinders the growth and proliferation of bacteria, thereby exerting its antibacterial effects.

It can be concluded that the C-lobe of lactoferrin plays a significant role in its antiviral and antibacterial actions since it seems to be largely involved in iron acquisition processes and consequently in preventing viral infections, interfering with viral fusion and protecting against drug-induced gastropathy. Additionally, the C-lobe exhibits antibacterial activity, making it a crucial component in lactoferrin’s multifunctional defense mechanisms.

## 2. Structural Characterization of Lactoferrin Interactions with Bacterial Proteins

### 2.1. Evolutionary Overview on Lactoferrin Receptors in Bacteria

Life began between 3.5 and 4.2 billion years ago in the primordial seas, which were rich in soluble iron, crucial for catalyzing redox reactions. This abundance of iron is believed to have contributed to its essential role in contemporary organisms. Photosynthesis, primarily performed by cyanobacteria, one of the earliest life forms, played a key role in this process. The oxygen produced by these bacteria reacted with iron, leading to the formation of iron oxides and a decrease in iron concentration in the seas. This event, known as the Great Oxidation Event, ultimately led to the accumulation of oxygen in the atmosphere and made iron a limiting nutrient in oceans.

As iron levels dropped, early prokaryotes evolved complex systems for iron acquisition and storage, including siderophores and ferritin. This evolution also impacted the sedimentation of iron in ocean floors. The significance of the existence of ancient prokaryotes on Earth is largely supported by stromatolites. Stromatolites are aggregates created by multiple species (including *Archaea* and *Cyanobacteria*) that exist both currently and in well-documented fossil records dating back to nearly 2 billion years ago. Such microbial communities, which include bacteria that produce siderophores, have been a consistent aspect throughout Earth’s history [37].

The adaptation of multicellular organisms to low-iron environments involved the development of iron-binding proteins like transferrins. Transferrin (Tf) is seen as an evolutionary response to the microbial communities and the challenges of iron acquisition. This protein originated from single-lobed ancestors, with the more complex bi-lobed variants evolving through gene duplication. Initially, transferrin homologues appeared on surfaces colonized by microbial communities for iron acquisition, later becoming integral to iron regulation in vertebrates. Transferrin, present on mucosal surfaces in humans and other vertebrates, has influenced evolutionary interactions between hosts and microbes.

These systems have been definitively identified in the *Pasteurellaceae*, *Neisseriaceae*, and *Moraxellaceae* families, and presumptively in others like *Alcaligenaceae*, based on bioinformatics analysis [38]. The more common bipartite receptor, consisting of transferrin-binding protein A and B (TbpA and TbpB) proteins, might have evolved from an intermediate single receptor stage that no longer exists. The evolutionary path likely entailed altering the siderophore receptor to facilitate iron removal from transferrin, similar to the role seen in the Tbp type A2 receptor protein. Nonetheless, only two species of bovine pathogens seem to possess TbpA2, and it attaches to the N-lobe of transferrin, indicating it may belong to a newer evolutionary branch. The TbpB protein possesses an extended anchoring peptide that allows it to extend beyond the cell surface and through the extracellular polysaccharide found in some species, thereby enhancing its ability to capture the iron-loaded form of Tf more effectively [39,40].

A study on primate transferrins [41] showed sequence variation at interaction sites with TbpA, indicating the TbpA–Tf interaction has been present for 40 million years of primate evolution. Since TbpA orthologues exist in chicken pathogens, this interaction might date back 320 million years, to the divergence of the Sauropsid (bird/reptile) and Synapsid (mammal) lineages [42].

It is thought that the gene duplication responsible for forming a bi-lobed precursor to Tf happened more than 580 million years ago. Following this, another duplication event took place less than 125 million years ago, giving rise to a different bi-lobed ferric binding glycoprotein, which is the progenitor of today’s lactoferrin. This novel gene product evolved to gain new functions, while the initial protein continued to regulate iron balance in the body independently [1]. Lactoferrin, notably present in colostrum and milk, plays a crucial role in iron sequestration. It is also found in various mucosal secretions like tears, saliva, nasal and bronchial secretions, vaginal fluids, and semen, and it is a major component in neutrophils’ secondary granules. The presence of lactoferrin receptors in certain bacterial lineages, which also possess transferrin receptors (like *Neisseriaceae* and *Moraxellaceae*), indicates that on mucosal surfaces bacteria might utilize lactoferrin as an iron source. However, lactoferrin receptors are notably absent in the *Pasteurellaceae* family. Lactoferrin might be a less reliable iron source as it is secreted in its apo (iron-free) form and is usually present at low levels on mucosal surfaces until a bacterial challenge occurs.

The high pI of lactoferrins initially made it challenging to identify and characterize lactoferrin receptor proteins as they tended to interact nonspecifically. To minimize these interactions, solid phase binding assays and affinity capture experiments were conducted under conditions of high pH and salt, which inadvertently led to missing some interactions. The first identification of the lactoferrin receptor in *N. meningitidis* by [43] revealed a single 100 kDa protein, now known as lactoferrin-binding protein A (LbpA). This finding was echoed in studies of other species [44]. The lipoprotein part of the receptor, LbpB, was identified later [45] when different affinity isolation conditions were used, though this increased the chance of nonspecific background. Like most transferrin receptors, the genes for lactoferrin receptor proteins, LbpA and LbpB, are arranged in an operon, with LbpB coming before LbpA [46,47,48]. In *M. catarrhalis*, a third gene with an unknown function is also part of this operon [49].

Beyond its iron-binding role, lactoferrin has evolved to have multiple additional iron independent functions. It can release cationic antimicrobial peptides from its positively charged N-terminal region. The presence of acidic amino acid-rich regions in LbpB, which protect against these peptides, suggests co-evolution with lactoferrin. Despite this, questions remain about the role of LbpB in iron acquisition, especially since *Neisseria meningitidis* can release it from its surface. The exact role of the lipoprotein component of transferrin and lactoferrin receptors in iron acquisition, particularly under in vivo conditions with limited iron, has not been fully explored.

LbpB is transported to the outer leaflet of the outer membrane in Gram-negative bacteria via numerous translocation and processing phases. Upon reaching its mature state, its integration in the membrane is facilitated by the addition of a palmitoyl group to the far N-terminal cysteine [50]. A notable feature of LbpB across diverse organisms is a region in the C-terminal lobe that can negate lactoferrin’s antimicrobial effects. The methods by which these proteins achieve this vary, reflected in the structural diversity of their C-terminal regions. Early research suggested that the sequestration of cationic antimicrobial peptides from lactoferrin is achieved by negatively charged amino acid stretches (Asp, Glu) in LbpB [51]. These regions are highly dynamic, making them difficult to crystallize and study structurally.

So far, structural knowledge of LbpB is limited to its N-terminal lobe, with successful crystallization from *Neisseria meningitidis* and *Moraxella bovis* [52,53]. However, in silico analysis of the C-terminal regions reveals significant variability. Human pathogens like *Neisseria meningitidis* and *Moraxella catarrhalis* have LbpBs with negatively charged regions, while livestock pathogens have larger domains with potential lactoferrin-binding capabilities. Sequence alignment shows high diversity in the C-terminal regions and less in the N-terminal regions, suggesting adaptation in response to lactoferrin’s evolving an-timicrobial functions [50].

The precise structure and mechanism of action of the C-terminal lobe of LbpB are not yet fully understood. Research is underway to investigate its role, but due to the structural variation, it is anticipated that LbpBs may not all operate in the same way. For example, in organisms such as *Neisseria meningitidis*, clusters of negative charges could attract and bind cationic antimicrobial peptides (CAMPs) through electrostatic forces. Conversely, in cases with larger domains, there might be inhibition of proteolytic breakdown or concealment of the antimicrobial areas of lactoferrin.

Further research is needed to understand how these Gram-negative bacteria use LbpB to counteract the mammalian innate immune system, with each organism potentially employing different strategies.

### 2.2. Characterization of Lactoferrin Interactions with Bacterial Membranes

The bacteriostatic activity of lactoferrin arises through its ability to deprive iron, an essential nutrient for cellular growth, from bacteria. The bactericidal mechanism of lactoferrins was first reported by [54]. The authors demonstrated that in a medium rich in iron, human lactoferrin could suppress the proliferation of *Vibrio cholerae* and *Streptococcus mutans*, yet it had no effect on the growth of *Escherichia coli*, and this antibacterial effect in the presence of supplemental iron. Arnold et al. therefore proposed, drawing on immunofluorescence research, that human lactoferrin attaches to bacterial cell surfaces. Indeed, the biological functions of human lactoferrin (hLf) are not solely attributable to iron binding but also to its ability to connect with various molecules.

Further studies have shown that bovine lactoferrin [55] and human lactoferrin [56,57,58,59] bind and release lipopolysaccharides (LPS) from gastric Gram-negative bacilli and demonstrated that lactoferrins can interact directly with the bacteria cell membranes. Therefore, interaction of hLf with bacterial outer membrane components such as LPS and porins is presumably important in the antimicrobial activity of hLf.

**Figure 2:**
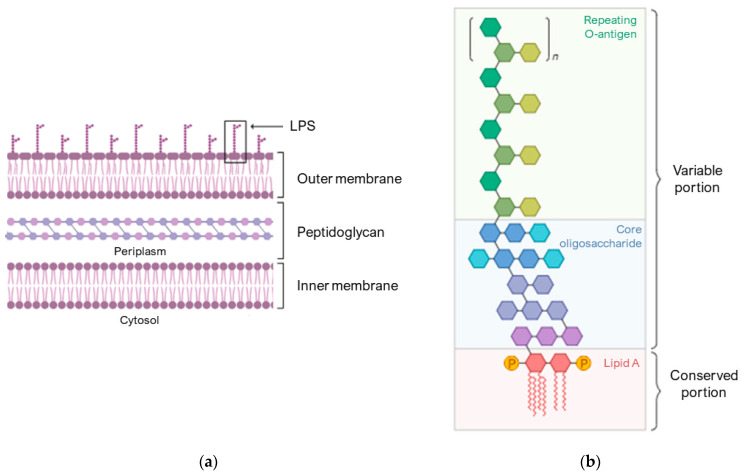
Compositions of the Gram-negative bacterial cell envelopes. (**a**) Structure of the asymmetric lipid bilayer. (**b**) The variable portion of LPS is composed of a repeating O-antigen and a core oligosaccharide. The conserved portion and the anchor to the membrane is lipid A. Image created with BioRender.

Figure 2a schematically shows the composition of the membrane of Gram-negative bacteria. The outer membrane is an asymmetric lipid bilayer with an inner and outer leaflet. The inner leaflet is rich in phospholipid; the outer leaflet is predominantly comprised of LPS (Figure 2b), which are composed of lipid A, a short core oligosaccharide, and an O antigen [60].

Binding of hLf to the lipid A portion of LPS [61] inhibits the priming of neutrophils for enhanced formyl-Met-Leu-Phe-triggered superoxide release [62] and might also account for the decreased production of cytokines after challenge with LPS [63]. Direct intermolecular interaction between hLf and human lysozyme (hLZ) [21] might contribute to the synergy between the antibacterial actions of these two proteins [64].

**Figure 3:**
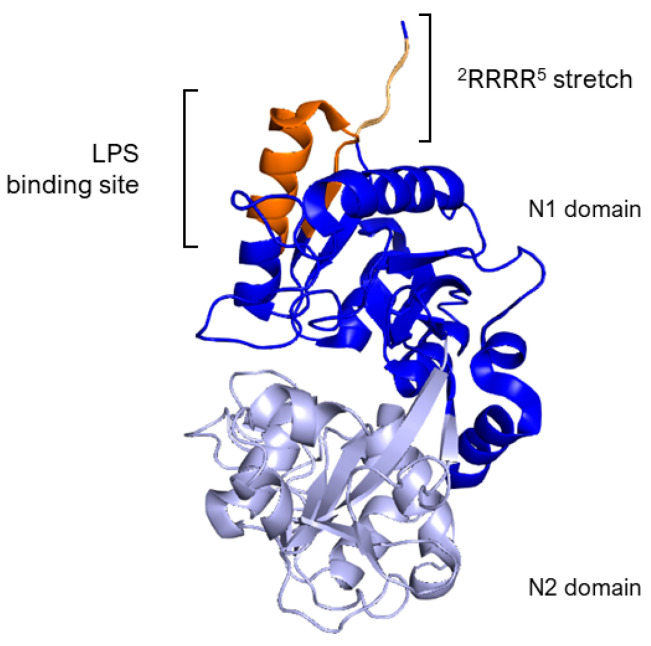
Side view of N1 (blue) and N2 (lavender) domains of Lf. The image highlights, in dark orange, the loop region of amino acids 20–37 corresponding to the LPS binding site of hLf. The ^2^RRRR^5^ stretch is in light orange. Image created with PyMOL Molecular Graphic System version 3.0 Schrödinger, LLC.

The essential segment for binding, identified as the Arg2–Arg3–Arg4–Arg5 stretch (^2^RRRR^5^), is found within the structure of human lactoferrin, as outlined in references [61,64]. Specifically, LPS binding site in hLf is situated within the loop region of the N-terminal domain, spanning amino acids 20 to 37, with a more precise focus on amino acids 28 to 34 (Figure 3). This particular loop region is also a feature of the sequence for the lactoferrin cleavage peptide, known as lactoferricin, which includes amino acids 1–49. In the case of bovine lactoferrin, the equivalent loop region stretches from amino acids 19 to 36 and constitutes the majority of the bovine lactoferricin sequence, as detailed in Section 5.1.

In the study by Elass-Rochard et al. [59], two LPS-binding regions were identified in hLf, with higher (K_D_ = 3.6 nM) and lower (K_D_ = 390 nM) binding affinity. This low-affinity binding site was identified by LPS-binding studies with the 51 kDa C-terminal tryptic fragment. However, such results provide no clear evidence for the existence of an LPS-binding site on the C-lobe.

In addition to the lipid A moiety of bacterial LPS, hLf also interacts with heparin, human lysozyme (hLZ), and DNA. The study by van Berkel et al. [64] explores how hLf from human milk (natural hLf) and N-terminally deleted hLf variants interact with those molecules. The research revealed that both iron-saturated and natural hLf effectively bound to all four molecules. However, removing the first two amino acids from hLf reduced its binding capacity by varying degrees to each molecule. Further deletions resulted in even lower binding efficiencies, with no binding observed in a variant missing the first five residues. The study concluded that a specific sequence of four arginine residues near the start of hLf plays a critical role in its ability to bind these molecules. An antibody targeting this region effectively blocked human lactoferrin interactions, underscoring the importance of this sequence in molecular interactions of the protein.

### 2.3. The Process of Iron Uptake Mediated by Membrane Receptors

**Figure 4:**
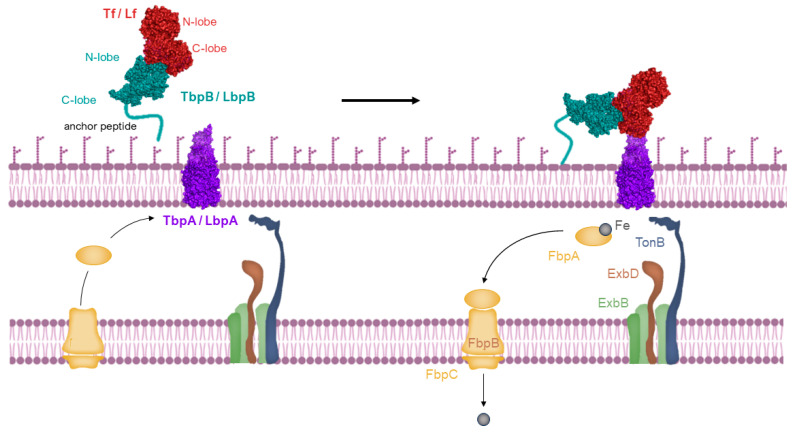
Iron is acquired from transferrin through a process where the long anchoring peptide of TbpB reaches out from the outer membrane’s surface to seize iron-laden transferrin (**top left**). TbpB then hands over this iron-rich transferrin to TbpA, initiating a reaction with TonB (**top right**). This interaction is fueled by energy from the ExbB-ExbD-TonB complex, enabling the iron’s transport through the outer membrane. Once separated from transferrin, the iron is handed over to the periplasmic iron-binding protein FbpA (Ferric-Binding Protein A), which moves it across the periplasmic space. It then interacts with a transport complex on the inner membrane, FbpB-FbpC, which utilizes ATP hydrolysis to move the ferric ion into the cytoplasm (**bottom right** and **left**). Image created with BioRender.

Host-specific transferrin and lactoferrin receptors were initially discovered in *Neisseria* species, pathogens of humans, as previously introduced [43,65,66], and were later found in other Gram-negative pathogens. These pathogens infect the upper respiratory or genitourinary tracts of mammals, including humans and animals [67,68]. The standard structure of the Tf receptor is bipartite, consisting of TbpA, an integral outer membrane protein dependent on TonB, and TbpB, a lipoprotein anchored to the surface (illustrated in the top left of Figure 4). TbpB, the surface lipoprotein, is designed with an N-terminal anchor peptide that enables it to extend from the outer membrane surface for the capture of iron-laden Tf [69,70]. This action facilitates the interaction of TbpB with TbpA, leading to the formation of a ternary complex with Tf and TbpA for iron extraction (shown in the top right of Figure 4). Unlike TbpA, which has equal affinity for both the apo- and holo- forms of Tf, TbpB significantly prefers the holo form. The domain separation in the C-lobe of Tf facilitates removal of iron, which is then transported across the outer membrane [71], a process powered by the TonB:ExbB:ExbD complex interaction (depicted in the bottom right of Figure 4). Following transport, the iron moves to the periplasmic ferric-binding protein A (FbpA), which conveys it to the FbpB and C inner membrane transport complex for cytoplasmic entry. FbpA is then recycled through the periplasm to engage once more with the TonB:ExbB:ExbD complex (shown in the bottom left of Figure 4).

Being a surface-exposed lipoprotein, TbpB’s selective capture of holo-Tf is hypothesized to function in the transfer of holo-Tf to the integral outer membrane protein TbpA. Notably, with the exception of *N. meningitidis* strain B16B6 or in TbpA mutants of *N. gonorrhoeae* [72,73], the necessity of TbpB for growth using Tf as an iron source under laboratory conditions is not observed. Consequently, the significance of TbpB in facilitating the delivery of holo-Tf to TbpA is presumed to be critical, predominantly under the more demanding growth conditions found in vivo.

While lactoferrin and transferrin receptors are identified in *Neisseriaceae* and *Moraxellaceae* families, their distribution in other species and roles in iron acquisition are less clear. Further, the specific roles of receptor proteins like LbpA and LbpB in different species, including their interaction with host proteins and impact on pathogenicity, remain areas of ongoing research.

The absence of a detailed structural definition for either LbpA or LbpB in complex with Lf leads to assumption based on comparisons with the structural homologs from Tf receptors system. It is likely that LbpA’s structure resembles TbpA, and LbpA probably interacts with the C-lobe of Lf, given the similarities in their interaction patterns. The LbpA is thought to share the iron transport mechanism with TbpA, suggested by the identity in their plug regions and a conserved motif EIEYE [74,75].

However, predictions about LbpB’s interaction with Lf and its role in iron acquisition are speculative due to limited structural data. The interaction between LbpB and Lf appears to be influenced by pH and salt levels [45], and LbpB is capable of providing protection against the antimicrobial activity of the human lactoferrin-derived peptide lactoferricin [38].

LbpB, when released from the bacterial surface, likely doesn’t contribute to iron acquisition in the same way as the TbpA–TbpB receptor. This suggests that LbpB may have evolved from a role similar to TbpB to a primary function of protection against cationic peptides, potentially altering its binding properties. The fact that many clinical gonococcal isolates lack Lf utilization, combined with the observed advantage of having a functional lbp operon in infection models, may imply different evolutionary pressures shaping LbpB’s role in bacterial survival and pathogenesis.

### 2.4. Crystalloghaphic and Cryo-EM Data of Lactoferrin-Binding Protein

Extensive studies on the identification and functional characterization of the *N. gonorrhoeae* and *N. meningitidis* Lbp gene product have been conducted [76,77,78,79] in order to shed light on the ever-scarcely characterized role of LbpA and B in Lf iron utilization.

However, despite numerous hypotheses regarding direct interactions between lactoferrin and target proteins, high-resolution structural data of such complexes remain scarce.

Preliminary sequence homology predicted a bi-lobed structure, and even though there is relatively little sequence identity with TbpBs, it likely shares an overall structural similarity with *A. pleuropneumoniae* TbpB structure [69].

Sequence alignments indicate that the barrel and the handle domains in N-terminal and C-terminal lobes retain a general conservation of their secondary structure elements. The alignment allows for the creation of a structural model for LbpB reported by Moraes et al. [69], and while the exact structure and characteristics of the surface-exposed loops remain uncertain, their positioning is probably accurate. A notable distinction between LbpBs and TbpBs lies in the two negatively charged areas found in the C-terminal lobe of LbpB.

**Figure 5:**
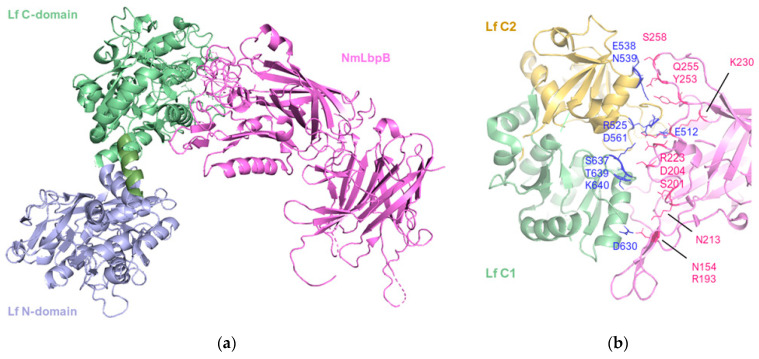
The 2.85 Å crystal structure of *N. meningitidis* LbpB (NmLbpB) in complex with human lactoferrin. (**a**) Orthogonal views of the complex with NmLbpB in violet and Lf C- and N- domains in green and lavender, respectively. The N-lobe of NmLbpB only interacts with the C-lobe of Lf along an extensive interface. (**b**) A zoomed-in view of the binding interface showing the interaction sites along an elongated surface covering both the C1 and C2 domains of Lf (colored in green and wheat respectively), with buried surface area 1760.8 Å^2^ (PDB: 7JRD). Image created with PyMOL Molecular Graphic System version 3.0 Schrödinger, LLC.

A significant advancement in the characterization of the crystal structure of *N. meningitidis* LbpB in complex with human lactoferrin was achieved by Yadav et al. [80], and it is reported in Figure 5.

The structure revealed a single complex, comprising a molecule of Lf with well-ordered N- and C-lobes, and a molecule of NmLbpB with an ordered N-lobe but a partially ordered C-lobe.

The overall configuration of the NmLbpB-Lf complex is L-shaped. The N-lobe of NmLbpB, encompassing residues 1–342, is divided into two domains: a handle domain at the N-terminus (residues 45–173) with four anti-parallel β-strands and an α-helix, and a β-barrel domain with eight strands (residues 174–342). On the other hand, the C-lobe of NmLbpB, spanning residues 359–718, exhibits notable disorder in the crystal structure, particularly in the region of residues 359–540 (handle domain) and 541–718 (β-barrel domain). Although the C-lobe β-barrel domain shares homology with the N-lobe, its handle domain differs, featuring a six-stranded β-sheet.

The structure of Lf also includes N- and C-lobes, each divided into two subdomains (N1, N2 for the N-lobe and C1, C2 for the C-lobe). In presence of iron, the subdomains of each lobe form a closed state, while in its absence, they adopt an open conformation [16,81]. Structural alignment indicated minimal changes in Lf upon binding with NmLbpB (RMSD of 1.3 Å) as both lobes were in closed conformations, consistent with the presence of iron in each lobe. The SAXS scattering curve calculated from the crystal structure closely matched the experimental SAXS curve obtained in solution.

The interaction between NmLbpB and Lf is mediated solely through the N-lobe of NmLbpB and the C-lobe of Lf. More specifically, the interface involves the residues D630, S637, T639, K640 of the C1 domain and E512, R525, E538, N539, D561 of the C2 domain; and residues N154, R193, S201, D204, N213, R223, K230, Y253, Q255, and S258 on the N-lobe of NmLbpB. Solid-phase binding assays confirmed that Lf interacts only with full-length NmLbpB or its N-lobe, but not with the C-lobe alone.

Cryo-electron microscopy (cryoEM) study, employed to ascertain the structure of the NgLbpB-Lf complex, revealed clear density for the side chains of both lobes of Lf and the N-lobe of NgLbpB, whereas the C-lobe of NgLbpB was almost indiscernible in the density map without significantly reducing the map contours.

Hints of the presence of a conserved binding interface between the Nm and Ng variants are provided by comparative structural alignment of the Nm and NgLbpB complexes with Lf. Moreover, a comparison between the cryoEM structure and the crystal structure of the NgLbpB-Lf complex show a difference of approximately 12° of the position of the N-lobe of Lf, suggesting a possible significant role of such variation in the function of LbpB.

In the same manuscript, analysis utilizing static small-angle X-ray scattering (SAXS) revealed that when isolated, NmLbpB tends to form aggregates in solution. In contrast, a stable, monodisperse complex is formed when NmLbpB is combined with Lf.

#### Evidence of Interactions with Porins

Porins are transmembrane proteins that create channels through the outer membrane of Gram-negative bacteria in order to facilitate the non-specific movement of hydrophilic solutes [60]. Hypothesis of antimicrobial activity of lactoferrin arise from evidence of bLf interacting with porins, in particular OmpF and OmpC [82,83].

Binding experiments were performed on both purified porins and porin-deficient *Escherichia coli* K12 isogenic mutants. Sallmann et al. [84] determined that lactoferrin binds to the purified native OmpC or PhoE trimer with molar ratios of 1.9 ± 0.4 and 1.8 ± 0.3 and K_D_ values of 39 ± 18 and 103 ± 15 nM, respectively. However, such strong binding has not been observed with OmpF, probably due to the fact that hLf binds to structural motifs that are present on OmpC but not on OmpF. Moreover, lactoferrin showed a preference for binding to strains expressing OmpC or PhoE. The study also revealed that lactoferrin amino acid residues 1–5, 28–34, and 39–42 interact with porins. Comparative sequence analyses have facilitated discussions on the interaction between lactoferrin amino acid residues and porin loops. Electrophysiological studies indicated that lactoferrin can inhibit OmpC but not PhoE or OmpF. Nonetheless, complete growth inhibition was only observed in the PhoE-expressing strain. These findings support the hypothesis that the antibacterial efficacy of lactoferrin might be partly attributable to its capacity to bind to porins, thereby altering the stability and/or permeability of the bacterial outer membrane.

Interactions with many mammalian cells target molecules, such as receptors [85,86,87,88], DNA [64], and proteoglycans [64,89,90,91,92], are mediated by the N-terminal sequence ^2^RRRR^5^ and a loop ^28^RKVRGPP^34^ of hLf. Additionally, it is theorized that the loop region ^39^KRDS^42^, known to inhibit platelet aggregation [93], contributes to the structure of the hLf receptor [87]. These critical areas are also components of human Lfcin, a peptide already introduced in Section 2.2 with antibacterial properties which is derived from the first 47 residues of hLf [94]. Furthermore, studies have indicated that sequences ^2^RRRR^5^ and ^28^RKVRGPP^34^ exhibit strong binding affinity for the lipid A portion of LPS [61,64,83,95]. This body of evidence points to the significance of residues 2–5, alongside loops 28–34 and 39–42 of hLf, in facilitating interactions with OmpC and PhoE porins.

## 3. Lactoferrin Interactions with Viral Proteins

The following section of the present review compiles the most significant scientific papers on the antiviral action of lactoferrin, providing a comprehensive examination of its efficacy against a variety of viral targets. Accompanied by Table 1, this review categorizes the research based on the type of lactoferrin studied (e.g., bovine, human), the viral targets (such as influenza, HIV, hepatitis), and the primary methods of exposure used to elucidate the hypothesized antiviral mechanisms.

The table highlights the versatility and potential of lactoferrin as an antiviral agent. This summary details the antiviral properties of lactoferrin as observed in various studies, focusing on its effects against a wide range of viruses, including HIV-1, HCMV, HSV-1 and 2, EBV, poliovirus, HCV, adenovirus, CHV, echovirus, EV71, influenza A, and SARS-CoV-2.

Initially, research by Harmsen et al. [96] demonstrated lactoferrin’s ability to prevent HCMV infection and reduce the cytopathic impact of HIV-1, with modifications enhancing its defense against HIV-1. Berkhout et al. [97] found modest inhibition of HIV-1 by lactoferricin, highlighting lactoferrin’s role in blocking HIV-1 entry by targeting both CXCR4 and CCR5 coreceptors. Groot et al. [98] showed lactoferrin’s capability to inhibit HIV-1 transmission from dendritic cells to T cells. Marchetti et al. [99] confirmed lactoferrin’s capability to inhibit HSV-1 infection, emphasizing its action in the initial stages of viral attachment.

Studies extended to metal-saturated lactoferrins demonstrated their efficacy in reducing HSV infection, with iron and manganese-saturated forms showing higher selectivity. Research indicated that lactoferrin impedes viral adsorption to cells by competing with virus receptor binding sites. For example, Ammendolia et al. [102] observed lactoferrin’s inhibitory activity against HSV-1 in GMK cellular models, further underscoring its potential as an infection treatment against herpes virion. Moreover, research on poliovirus infection indicated that all forms of lactoferrin, particularly zinc-saturated lactoferrin, could prevent viral replication at various infection stages.

Investigations into lactoferrin’s interaction with HCV highlighted its specific binding to envelope proteins E1 and E2, inhibiting HCV infection in hepatocytes. Liao et al. [109] assessed the effects of recombinant and native camel lactoferrin on HCV, showing inhibition of entry and amplification. Picard-Jean et al. [110] studied human lactoferrin’s antiviral activity against HCV, revealing its capacity to bind and neutralize the virus and inhibit replication.

Pietrantoni et al. [111] and subsequent research have illuminated the antiviral properties of bovine lactoferrin across a spectrum of viruses. Specifically, bLf binds to adenovirus proteins, notably the penton base, and is implicated in the infection process of species C human adenoviruses (HAdVs) by attaching to the hexon protein, facilitating virus entry into cells through unknown receptors. Similarly, the interaction with canine herpesvirus (CHV) and echovirus demonstrates its capacity to inhibit viral infection and replication, either by preventing CHV infection in cells or by disrupting echovirus’s entry pathway. Research also highlights the protective role of bovine lactoferrin against enterovirus 71 (EV71) infection through its ability to bind to viral and cell components, including the VP1 protein, indicating a broad antiviral mechanism that includes blocking virus–cell interactions. More recent findings by Superti et al. [36] explore interaction of bLf with influenza A virus hemagglutinin, pointing towards its potential in preventing infection by stabilizing hemagglutinin at low pH, inhibiting fusion peptide activity and offering a novel target for anti-influenza therapeutics.

From the studies reported and the subsequent results obtained, a broad picture arises concerning the viral strains considered, the cellular targets, and the methods of exposure. What emerges from the experimental results is the ability of lactoferrin to bind to viral capsid proteins or to hinder the infection by occupying cellular membrane receptors. Research articles [116,117] rely on computational predictions, and this approach is discussed in more detail in the following section of the present manuscript.

Such considerations emphasize the wide therapeutic potential of lactoferrin, and they lay the groundwork for future research directions in the development of lactoferrin-based therapeutics.

## 4. Computational Approaches for the Investigation of Lactoferrin Activity against SARS-CoV-2 Infection

Lactoferrin is also a valuable substance for generating various peptides that exhibit antimicrobial, immunomodulatory, and antihypertensive properties [118]. In particular, it has been demonstrated that lactoferrin exhibits antiviral activity against the virus, especially during its early infection stage [119]. More specifically, it has been shown that lactoferrin has in vitro antiviral efficacy against SARS-CoV, which is in close relation, genomically, with SARS-CoV-2 [120]. In this scenario, one of the possible solutions to prevent the infection of the SARS-CoV virus is the inhibition of SARS-CoV-2 main protease (Mpro), a coronavirus protein that has been considered as one among many drug targets. In this context, in Zhao et al. (2022) work lactoferrin from *Bos taurus* L. was hypothesized to be a good candidate for interaction with the Mpro protease. The bioactivity, water solubility, and ADMET properties (absorption, distribution, metabolism, elimination, and toxicity) of the generated peptides were forecasted using diverse online tools. Molecular docking and molecular dynamic simulation were employed to examine the molecular interactions between Mpro and the peptides. Here, Zhao et al. [121] conducted in silico analysis on B. taurus lactoferrin to identify a potential SARS-CoV-2 inhibitor, selecting specific lactoferrin-derived peptides as potential inhibitors against the main protease of SARS-CoV-2.

Among the analyzed peptides, GSRY emerged as the most promising, boasting a bioactivity score of 0.5381 and favorable water solubility. ADMET analysis indicated a 21.72% absorption rate for the GSRY peptide. Notably, GSRY exhibited binding to Mpro through six conventional hydrogen bonds, seven carbon hydrogen bonds, one charge interaction, and one pi-alkyl interaction. Furthermore, their binding overlapped with the inhibitor N3 at critical residues (HIS163, GLY143, GLU166, GLN189, and MET165), crucial for successful Mpro SARS-CoV-2 binding. Molecular dynamic simulation affirmed the stability of the Mpro/GSRY complexes. In conclusion, the authors suggest that GSRY holds potential as a novel inhibitor for SARS-CoV-2. For these reasons, lactoferrin can also be a valuable material for the production of peptides that could potentially be used as Mpro inhibitors to hinder the replication of SARS-CoV-2.

In another study [122], computational analysis revealed the interaction between lactoferrin and transferrin receptor 1, indicating a diverse mechanism of action for lactoferrin. Protein-protein molecular docking simulations were carried out using the CLUSPRO method to explore the interaction between TfR1 and Lfs [123], considering in particular different variants of SARS-CoV-2. In addition, molecular dynamics simulations with standard approaches were performed for Spike and lactoferrin proteins.

Molecular docking simulations between bLf and the Alpha, Beta, Delta, and Omicron variants of the Spike protein reveal a consistent binding pose where the bLf structure interacts with the RBD domain in the up conformation. In all four docking simulations, the top three solutions, identified through clustering, represent over 60–70% of the total generated complexes and align with the binding pose established in a previous work [124].

Utilizing the initial solutions from the docking experiments, the authors conducted four 100 ns classical MD simulations to assess complex stability, persistent interactions, and the ability of bLf to engage with all Spike variants, regardless of the number and position of mutations. The simulations show that the interaction between the two proteins remains stable over time, with the two interfaces relatively close. MM/GBSA analyses confirmed a strong affinity of bLf for the Spike glycoprotein, with interaction energies of −36.2, −69.1, −46.4, and −45.8 kcal/mol for the Alpha, Beta, Delta, and Omicron Spike variants, respectively. Notably, the MM/GBSA results revealed a shift in the energy contribution to the binding energy, transitioning from the Van der Waals term for the Alpha variant (as observed in the Wuhan isolate) to the polar solvation term for the Omicron variant [124]. This indicates that, despite similar orientations during recognition, the interactions defining the complexes vary significantly among the four studied variants. These findings lead us to propose that bLf is likely to maintain its capacity to bind to the surface of the Spike glycoprotein, irrespective of the mutations identified in the emerging variants of concern. In conclusion, the authors’ results highlight the existing binding between Lf and Spike, confirming the ability of lactoferrin to hinder Spike-mediated pseudoviral entry and Spike-induced iron dysregulation. Therefore, the use of bovine lactoferrin, which is already available as a nutraceutical, in contrasting COVID-19 infection appears to offer promising potential as a complementary approach to standard therapies.

Analyzing the interaction between the SARS-CoV-2 Mpro protease and lactoferrin is just one of the strategies explored in recent years to counteract the infectious effects of the novel coronavirus. The authors conducted a computational study focusing on the early stages of infection, specifically the attachment and entry of the virus into the host cell, where lactoferrin can interfere with the virus–host interaction without the need for internalization [116]. The authors employed a recently developed computational method based on 2D Zernike polynomials to shed light on the molecular mechanisms supporting the antiviral action of human lactoferrin against SARS-CoV-2. This method allows for an efficient, fast, and unsupervised description of local geometrical shapes, facilitating easy comparison between different regions of molecules [125]. The study particularly focused on the attachment and entry stages, investigating whether lactoferrin could compete with the virus in binding to different components: (i) the spike protein, (ii) the ACE2 receptor, and (iii) sialic acid, a component implicated in the attachment of SARS-CoV-2 to the host cell, as initially demonstrated computationally [126,127] and subsequently confirmed experimentally [128,129,130].

An exploration of the interaction between lactoferrin and the primary SARS-CoV-2 protein receptor, ACE2, revealed a hot-spot region oriented toward the membrane, suggesting potential interaction. Analysis of the three membrane proteins on the virus envelope indicated possible interacting regions in E and M proteins, buried under normal conditions. The spike protein displayed two robust hot spots, with the most complementary region located in the C-terminal region involved in the spike–ACE2 interaction. This region, distant from known spike glycosylation sites, suggests a possible competition between ACE2 and lactoferrin for binding to the SARS-CoV-2 spike, providing insight into the observed antiviral action.

Another computational study was carried out to analyze the molecular interaction mechanisms that would lead to a reduction of the infection during its early stages [131]. Pelargonium sidoides extracts and lactoferrin, both noteworthy natural anti-inflammatory and antiviral agents, possess the potential to disrupt the initial phases of SARS-CoV-2 infection. Employing molecular docking and molecular dynamics simulation approaches, the authors investigated interactions between Pelargonium sidoides compounds, lactoferrin, and components of SARS-CoV-2. Computational analyses, mainly based on molecular dynamics simulation and energy estimation, indicate that Pelargonium sidoides extracts can interact with lactoferrin without causing alterations to its structural and dynamic properties. Moreover, Pelargonium sidoides compounds appear capable of interfering with the Spike glycoprotein, the 3CLPro, and the lipid membrane, potentially influencing the functional properties of proteins within the double layer. These findings suggest that Pelargonium sidoides may disrupt the mechanism of SARS-CoV-2 infection, particularly during its early stages.

## 5. Lactoferrin-Derived Peptides and Chimera

**Figure 6:**
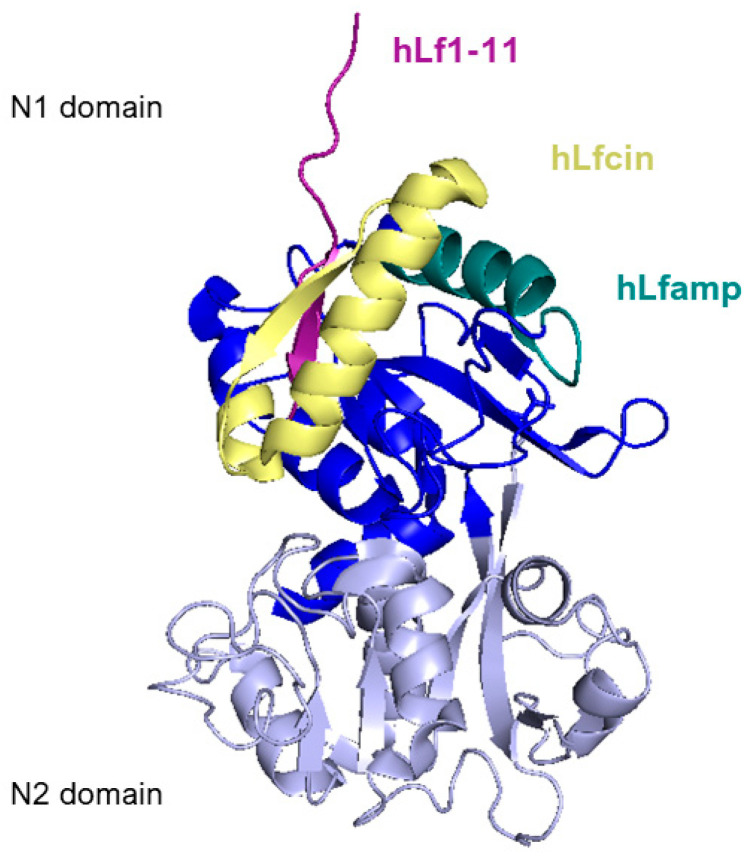
Side view of N1 (blue) and N2 (lavender) domains of human lactoferrin. Lactoferricin peptide is represented in yellow, lactoferrampin is in dark teal, and Lf1-11 is in purple. Image created with PyMOL Molecular Graphic System version 3.0 Schrödinger, LLC.

Figure 6 reports a side view of the N-lobe of human lactoferrin highlighting the positions of the structure of the main lactoferrin-derived peptides that will be described in the following section.

### 5.1. Lactoferricin

Lactoferricin (Lfcin), a peptide obtained through the enzymatic breakdown of lactoferrin with pepsin, marked a significant discovery in antimicrobial research. Initially, lactoferrin was recognized for its antimicrobial qualities due to its capacity to bind iron. However, it was later discovered that lactoferricin possessed even stronger antibacterial properties than its precursor, sparking a wide range of studies on both natural and synthetic peptides [132,133,134]. These investigations have enhanced the understanding of the antibacterial mechanisms at the molecular level, highlighting that the antimicrobial attributes are concentrated in the protein’s highly basic N-terminal regions, which do not participate in iron binding [94,95].

Lactoferricin was first isolated from bovine lactoferrin by Tomita et al. [135], showing superior or comparable antibacterial effectiveness against various bacteria, even those resistant to lactoferrins. Bovine and human lactoferricins are the most well-known, but peptides based on lactoferrins from other species like goats, mice, and pigs have also been identified or synthesized. Bovine lactoferricin is notably more effective against Gram-positive bacteria compared to Gram-negative ones.

Lactoferricins comprise several hydrophilic and positively charged amino acids encircling a hydrophobic core (specifically, the hydrophobic amino acids: Phe1, Cys3, Trp6, Trp8, Pro16, Ile18, and Cys20), which characterize their amphipathic and highly cationic nature. The amphiphilic configuration of lactoferricins in a solution enables their interaction with biological membranes and anionic elements present in bacterial outer membranes or cell walls, such as LPS or lipoteichoic and teichoic acids. Research by Umeyama et al. [136] revealed that bovine lactoferricin exhibits a higher affinity for acid phospholipids compared to neutral phospholipids. This specificity extends to interactions with bacterial cell membranes, distinguishing them from eukaryotic cell membranes. Moreover, lactoferricin demonstrates even greater affinities for acid phospholipids than their native lactoferrins, potentially elucidating their enhanced antibacterial properties.

Furthermore, it has been found to form pores in acid phospholipid membranes and cause deformation and lysis of *Staphylococcus aureus* cell walls [137], though its antibacterial mechanism against Gram-negative bacteria does not involve cell lysis.

The non-lytic antibacterial strategy of lactoferricin involves penetrating bacterial cells to target intracellular processes, beginning with interactions at the bacterial membrane or cell wall to enable peptide entry. Both bovine and human lactoferricins bind to LPS on Gram-negative bacteria, facilitating LPS release. An alternative mechanism proposed by Farnaud et al. [138] involves a two-step process, where the peptide’s positive amino acids disrupt the outer membrane structure before tryptophan residues interact with lipid A, enabling membrane penetration.

**Figure 7:**
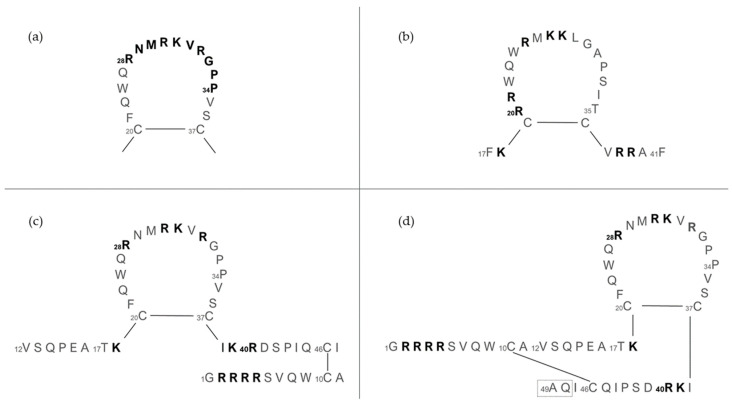
The amino acid sequence of lactoferrins and lactoferricins. (**a**) Loop region in the N-terminal lobe of human lactoferrin. Bovine lactoferricin (**b**) and human lactoferricin, originally defined with 47 amino acids (**c**) and later with 49 amino acids (**d**). Amino acids represented in bold are involved in bacterial binding; the dashed box in (**d**) reports two additional amino acids over (**c**). Image adapted from [140].

The structure and length of lactoferricins, cleaved from the N-terminal region of lactoferrin, vary across mammals, affecting their antimicrobial efficacy. For example, bovine lactoferricin, which consists of 25 amino acids from bovine lactoferrin’s N-terminus, displays a cyclic structure that enhances its antibacterial activity by allowing stronger and deeper membrane interactions compared to its linear counterpart. Human lactoferricin, first identified with 47 amino acids by Bellamy et al. [94], was later reported to contain 49 amino acids [139], with proposed cyclic structures similar to its bovine counterpart, indicating variations in lactoferricin structures and their antimicrobial impacts (Figure 7).

The crystallographic structure of lactoferricins has also been investigated by Hunter et al. [139].

**Figure 8:**
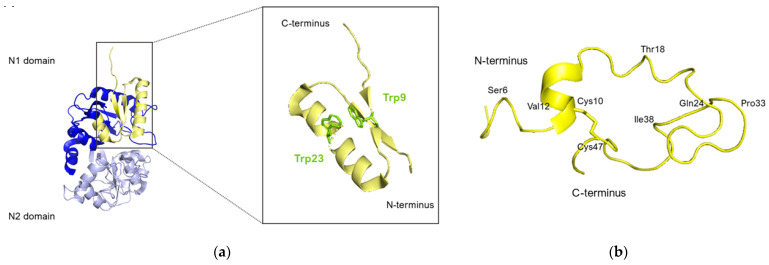
Human lactoferricin structure. (**a**) Lfcin isolated from the crystal structure of hLf. N1 and N2 domains of hLf are represented in blue and lavender respectively, Lfcin structure is represented in yellow. The Trp side chains are highlighted in green in the zoomed-in inset. (**b**) Ribbon representation of the average energy-minimized structure of hLfcin in aqueous solvent. The structural calculations of hLfcin in aqueous solution indicated a well-defined structure for regions of the peptide. These regions primarily involved Ser6 to Val12, Thr18 to Gln24, and Pro33 to Ile38. This structure shows a helical region for Pro15 to Thr18, and the coiled backbone continues to Gln24. A turn at Lys29 and Val30 leads Val30 to Cys37 back in an anti-parallel alignment to Cys20 to Gln24. However, except for the disulfide bridge from Cys10 to Cys47, there is no close association between Gly1 to Lys19 and Ile38 to Ala49 (figure adapted from [140], PDB: 1Z6W). Image created with PyMOL Molecular Graphic System version 3.0 Schrödinger, LLC.

Nuclear magnetic resonance (NMR) analysis revealed that bovine lactoferricin (bLfcin) adopts a secondary structure in water that slightly deviates into an antiparallel β-sheet configuration. This structure is distinct from its parent bovine lactoferrin, where the lactoferricin segment (amino acids 12–29) assumes an α-helical structure according to X-ray analysis. Furthermore, the research conducted on the 3D structure of human lactoferricin in an aqueous environment, utilizing a membrane-like solvent composed of dodecylphosphatidylcholine and sodium dodecyl sulfate, indicated a transition in human lactoferricin. Specifically, in the segment from Gln14 to Lys29, it initially forms an early-stage coiled helix in water, but demonstrates helical characteristics when in the membrane-mimicking solution (Figure 8).

However, Chapple et al. [141] found that the functional segments of human lactoferrin and lactoferricin take on a β-strand formation, as opposed to an α-helix, in the presence of LPS. In a similar manner, Farnaud et al. [142] through computer modeling, identified two β-strands connected by a pronounced turn in the N-terminal structure of human lactoferrin, diverging from an α-helical configuration. These structural variances could be key in understanding the enhanced antimicrobial capabilities of lactoferricins compared to their original lactoferrin form. The β-sheet structure of lactoferricins seems to be more effective in initial interactions with membranes and LPS than the α-helical structure seen in native lactoferrins. Moreover, Pei et al. [143] indicated that bovine lactoferricin undergoes structural changes in response to ionic strength and hydrophobic effects, which enhance its antibacterial properties. The superior antibacterial effectiveness of bovine lactoferricin compared to its human counterpart is believed to be due to variations in the distribution of charges around their hydrophobic cores.

Several studies have revealed that even smaller fragments of lactoferricin sequences show some antibacterial activity, such as the 15-amino-acid fragment (amino acids 17–31) [138,144,145,146,147]. It appears that the region of 11 amino acids from amino acids 20 to 30 in bLfcin is essential, with this region showing great antibacterial activity.

The antibacterial efficacy of lactoferricins is influenced by pH and various ions like Ca^2+^, Mg^2+^, Na^+^, and K^+^. Their activity can be reduced by both cations and anions, a phenomenon observed with other antimicrobial peptides as well.

### 5.2. Lactoferrampin

Another segment within the N1-domain is lactoferrampin (Lfampin), which shares typical features of antimicrobial peptides, like the presence of positively charged residues and a hydrophobic domain containing tryptophan, crucial for membrane insertion. A synthesized peptide corresponding to bovine lactoferrin residues 268–284 (Figure 6), Lfampin, shows broad-spectrum antimicrobial activity without any hemolytic activity at its antimicrobial working concentration, differing slightly from the activity of another domain of lactoferricin [148].

According to van der Kraan et al. [148], Lactoferrampin seems to be effective against various bacteria but ineffective against others, including *Porphyromonas gingivalis* and *Streptococcus sanguis*. This peptide doesn’t cause hemolysis at effective concentrations. Using the ‘PeptideCutter’ option of the ExPASy Proteomics Server, Bolscher et al. [149] predicted cleavage sites of bovine lactoferrin with site-specific endoproteinases such as ArgC (clostripain), AgrN, and ArgC/AgrN for the release of lactoferrampin and three other peptide fragments: f(259–284), f(265–296), and f(265–284). All four of these showed antibacterial activities against *E. coli*.

A human variant of lactoferrampin, corresponding to amino acids 269–285 in human lactoferrin, was also synthesized [150]. However, it was initially ineffective against *E. coli* and *S. sanguis*. Alterations to this human lactoferrampin, such as changing an amino acid or adding a lysine residue, eventually led to the inhibition of bacterial growth.

### 5.3. Lf1-11

Lf1-11, as its name suggests, is the N-terminal peptide of lactoferrin, comprised of the first eleven residues of the molecule (Figure 6).

This peptide has been shown to be highly effective against five multidrug-resistant *Acinetobacter baumannii* strains [151], methicillin-resistant *Staphylococcus aureus* [152,153], and various *Candida* species [154,155].

The effectiveness of Lf1-11 as an antimicrobial is primarily due to its initial two arginine residues at the N terminus [156]. This was further supported by Nibbering et al. [157], whose study reported that peptides lacking these arginines (R2–R4) were less effective. Moreover, lactoferrin variants without the first five N-terminal residues exhibited diminished ability to bind to bacterial lipopolysaccharides [64].

Stallmann et al. [158] conducted a study focusing on the use of human Lf1-11 for prophylactic treatment in a rabbit femur infection model, discovering that it notably hindered the progression of osteomyelitis. The ability of Lf1-11 to mitigate osteomyelitis is linked to its mechanism of causing mitochondrial damage, where extracellular ATP plays a significant yet not exclusive role. The peptide’s effectiveness against candida is dependent on its induction of calcium uptake by mitochondria.

The potential of the peptide to boost the host’s innate immune defense against infections was suggested, highlighting Lf1-11’s value in the development of a new treatment option for microbial infections, especially in patients with weakened immune systems, as noted in the study by Van der Does et al. [159]. Lf1-11 targets myeloperoxidase within monocytes for its immunomodulatory effects, binding and inhibiting it, as revealed through molecular modelling studies. These findings emphasized the critical nature of the first two arginines and a cysteine at position ten, showing peptides missing these residues had reduced efficacy in myeloperoxidase binding [160]. A comparative analysis of Lf1-11 across six species indicated amino acid sequence variations [161], with human Lf1-11 containing three arginines (R2–R4) unlike peptides from other species which feature only one arginine (R3) and often have lysine in place of R4, still maintaining the peptide’s positive charge. The presence of hydrophobic residues V6 and W8 across species underlines their importance in the peptide’s structural and functional integrity.

### 5.4. Other Lactoferrin-Derived Peptides with Antimicrobial Activities

The investigation of lactoferricins instigated numerous studies on the enzymatic breakdown of bovine lactoferrin, resulting in the extraction of a plethora of novel lactoferrin-derived peptides exhibiting antibacterial properties against both Gram-positive and Gram-negative bacteria. Pepsin was predominantly employed for enzymatic hydrolysis, although the experimental parameters in various studies often diverged from the original methodology employed by Tomita et al. [135]. Consequently, Dionysius et al. [162] succeeded in isolating three peptides from bovine lactoferrin. Peptides I and II demonstrated antibacterial efficacy against several pathogenic and spoilage-causing microorganisms, whereas peptide III exhibited reduced activity. Subsequently, Recio et al. [163] adopted two ion-exchange chromatographic techniques to scrutinize pepsin hydrolysates of bovine lactoferrin, isolating five peptides. Among these, peptides 2 and 4 were effective in inhibiting the proliferation of *Micrococcus flavus*. Later, Kim et al. [164] unveiled the inhibition of *Pseudomonas syringae* growth by a novel peptide from bovine lactoferrin, again via pepsin-mediated enzymatic hydrolysis. This decameric peptide, originating from the N-terminal region of bovine lactoferrin and spanning amino acids 308 to 317, was distinct from bLfcin.

Alternative enzymes have also been utilized for the proteolytic cleavage of lactoferrin. When employing trypsin, papain, and bacterial enzymes for cleavage, the resulting hydrolysates typically exhibited diminished antibacterial activities compared to those obtained with pepsin. Lizzi et al. [165] utilized trypsin for bLf hydrolysis, revealing that the complete hydrolysate possessed equivalent antimicrobial efficacy against tested Gram-positive and Gram-negative bacteria as the intact lactoferrin. However, peptides smaller than 5 kDa showed heightened bacterial growth inhibition compared to native lactoferrin, while larger peptides demonstrated no inhibitory effect, indicating that only the smaller peptides maintained antibacterial properties. Similarly, Rastogi et al. [166] conducted trypsin-mediated hydrolysis of bovine lactoferrin, isolating three peptides which, in contrast to the native protein, exhibited increased activity against Gram-negative bacteria. Hoek et al. [167] employed recombinant chymosin for enzymatic cleavage of bovine lactoferrin, producing four peptides with antimicrobial activities superior to native lactoferrin, including one with the amino acid sequence of bovine lactoferricin.

As observed with lactoferricin, all peptides derived from enzymatic hydrolysis were cationic and located in the N-terminal region of the bovine lactoferrin molecule. Notably, none of these peptides associated with lactoferricin contained any amino acids implicated in iron binding.

### 5.5. Lactoferrin-Chimera

The creation of a novel peptide, named lactoferrin-chimera, was achieved by fusing two segments: lactoferricin 17–30 and lactoferrampin 265–284. This fusion resulted in a peptide chain comprising 35 amino acids. This new molecular entity exhibited a heightened antimicrobial ability towards a range of Gram-positive and Gram-negative bacteria, surpassing the effectiveness of either lactoferricin 17–30, lactoferrampin 265–284, or their combination [149,168,169,170]. Additionally, lactoferrin-chimera has shown promising synergistic effects when used alongside various antibiotics against *Vibrio parahaemolyticus* strains resistant to multiple drugs [168]. Remarkably, lactoferrin-chimera also proved effective in inhibiting the growth of certain *Burkholderia pseudomallei* strains, which were resistant to the preferred antibiotic, ceftazidime [171].

Additional studies also indicate that these three peptides, including lactoferrin-chimera, interact with dimyristoylphosphatidylglycerol liposomes when used as a model for bacterial membranes. The interaction strength was highest with lactoferrin-chimera and lowest with lactoferricin [172,173]. Circular dichroism spectroscopy analysis has shown that lactoferrin-chimera and lactoferrampin 265–284 share an α-helix structure, while lactoferricin 17–30 has a β-turn structure [173]. The α-helix in lactoferrin-chimera, which resembles the spatial structure in native lactoferrin (known for weaker antibacterial activity compared to lactoferricin and lactoferrampin), suggests that the enhanced antibacterial properties of lactoferrin-chimera are likely due to its unique mechanism. Its high positive net charge (+12) facilitates binding to bacterial membranes, leading to their destabilization and permeabilization. Furthermore, lactoferricin 17–30 and lactoferrampin also demonstrated the ability to penetrate or translocate into the bacterial membrane of *S. pneumoniae* [167,168,169].

## 6. Lactoferrin Interaction with Plasma Proteins and Human Cell Receptors

Numerous investigations have delved into the intricate interplay among metalloproteins, specifically myeloperoxidase (MPO), ceruloplasmin (CP), and lactoferrin, pivotal in the regulation of inflammation and oxidative stress in vertebrates [174,175,176]. The studies further elucidated the nuanced interactions between CP, Lf, and MPO, portraying them as interconnected components operating synergistically to alleviate oxidative and halogenative stress associated with inflammation. Key observations highlighted the selectivity of CP’s interaction with both exo- and endogenous Lf in the bloodstream [177,178] and revealed the inhibitory potential of plasma CP on MPO, underscoring their collaborative efforts to counteract the detrimental effects of inflammation. Despite various physicochemical characterizations of these interactions, detailed high-resolution structural data at the single-molecule level remain limited. Noteworthy studies utilized high-resolution AFM to visualize individual MPO, CP, and Lf molecules, outlining the morphology of MPO-CP and Lf-CP complexes while confirming the absence of direct contacts between MPO and Lf [179]. The collective interpretation from these low-resolution data suggests that, in the presence of all three proteins, the molecular assembly may assume a sequential pentameric structure containing CP2Lf2MPO, where in CP and Lf exert modulatory effects on MPO activity. In a separate study, the SAXS approach not only established the stoichiometry of the CP:Lf complex in solution but also proposed a model for the mutual arrangement of the two proteins [180]. Rigid-body modeling of the complex, using the crystal structures of the partners, suggested that the C-terminal region of CP is involved in the interaction with Lf, indicating its role in binding to the N-terminus of Lf. This finding aligns with data on Lf, which identified the N-terminal ^2^RRRR^5^ stretch as the most probable candidate for interaction with CP [181].

As a last observation, it is of interest to comment on the controversial and still debated putative interaction between Tf and CP as compared to the demonstrated Lf:CP interaction [182]. According to most recent observations, CP would indeed bind Tf but only in the presence of Zn cations [183]. The molecular details of the interaction were suggested by a computational modeling for the complex between Zn(II)-bound CP and apo-Tf in which CP domains 4 and 6 fit perfectly into the opened structure clefts in the N- and C-lobes, respectively, in which two molecules of CP and one molecule of apo-Tf form a tightly bound trimer held together by the presence of Zn cations.

The search for a possible lactoferrin receptor in human cells has prompted numerous investigations that ultimately led to the identification of specialized lectins expressed in intestinal epithelia that are normally involved in bacterial lipopolysaccharide recognition [184,185]. In particular, human intelectin-1 (hIntL-1) has been reported to bind lactoferrin, suggesting that hIntL-1 could recruit lactoferrin to microbial cell surfaces for cell killing [186,187]. Binding data are based on affinity chromatography studies coupled to ELISA testing. The apparent affinity measured for the hIntL-1 trimer was, however, rather weak for a specific protein–protein interaction (K_D_ of ∼500 nM), and direct structural data are not available as yet. These potentially interesting data still need an in-depth analysis of the structural basis for their interactions. Along the same line, other proposed Lf receptors have been suggested, including LDL and TOLL-like receptors, but in all cases, structural evidence and thermodynamics of the interactions with Lf are not yet available [188].

## 7. Conclusions

In the study presented here, we investigated the foundational structures guiding the intricate relationships between lactoferrins and their target molecules, pivotal for effective therapeutic outcomes. Lactoferrins, along with their derived peptides, are renowned for their extensive range of therapeutic potentials, manifesting antiviral, antibacterial, immunomodulatory, anti-inflammatory, anti-anemic, neuromodulatory, and even anticancer properties. These properties are underscored by their significant clinical applications, as evidenced by the 215 clinical trials documented on ClinicalTrial website. www.clinicaltrials.gov (accessed on 27 February 2024). Among these, 25 trials are actively recruiting participants, while more than 100 have reached completion. Notably, 55 studies have focused on milk lactoferrin’s impact on pediatric morbidity related to bacterial infections and immune disorders. Despite these advancements, the molecular mechanisms driving these therapeutic effects remain largely unclear, predominantly relying on empirical associations. In recent years, there has been a concerted effort to elucidate the actions of lactoferrin at the structural level. A notable breakthrough in this endeavor was the determination of the first structure of a complex that included a lactoferrin molecule with well-defined N- and C-lobes, in conjunction with a molecule of NmLbpB. This structure, described in Section 2.4, is particularly promising for the development of inhibitors targeting the interaction crucial for iron uptake by various pathogenic bacterial species. At present, sites in the lactoferrin structure that are responsible for the manifestation of antiviral activities have only been inferred by computational biology methods, as outlined in Section 4. Direct observation of lactoferrin’s structure in complex with viral epitopes is eagerly anticipated to elucidate true mechanisms of action.

Furthermore, successful clinical applications, such as treating different types of anemia, have been supported by experimental findings rooted in cell or tissue biology, providing limited insight into the structural biology governing these actions. Similarly, significant therapeutic actions of lactoferrin-derived peptides, particularly against invasive fungal diseases, rely on robust experimental evidence from in vitro cell culture and tissues, yet understanding the molecular determinants governing interactions with target receptors lags behind.

Given this context, it is projected that a thorough understanding of the structural basis of lactoferrin’s interactions with its molecular partners will greatly enhance therapeutic applications and emerge as a fundamental aspect of lactoferrin’s medicinal chemistry. This deeper comprehension is expected to pave the way for novel therapeutic strategies and interventions, further solidifying the role of lactoferrin and its derivatives in the realm of therapeutic sciences.

## Figures and Tables

**Table 1:** List of the most relevant research papers that report in silico, in vitro, and in vivo experiments where inhibitory effects in viral infection and/or proliferation caused by the presence of Lfs of different origins have been observed.

Lactoferrin	Target Virus	Target of Exposure	Method of Exposure	Antiviral Activity	Reference
hLf/bLf	HIV-1	MT4 cells	MTT assay Time-of-addition assay	Interference with viral adsorption and proliferation	[96]
bLf/bLfcin	HIV-1	SupT1 T/U87-CD4 cells	Preincubation of cells with Lf MTT assay	Blocking of HIV-1 variants that use CXCR4 and CCR5 coreceptors	[97]
bLf	HIV-1	Dendritic cells	Time-of-addition experiments with single-cycle transmission assay Preincubation of cells with Lf	Inhibition of DC-mediated capture and transmission of HIV-1 and bLf-resistant HIV-1 through binding observed between Lf and DC-SIGN receptors	[98]
hLf/bLf	HCMV	Human fetal lung fibroblast cells	MTT assay Time-of-addition assay	Receptor-mediated binding observed to the target cell membrane	[96]
hLf/bLf	HSV-1	Vero cells	Particle agglutination assay Immunofluorescence assay ELISA assay	Inhibition of viral adsorption by blocking cellular receptors and glycans (LDL and HS) and/or viral attachment proteins (Lf in both apo- and holo-form)	[99]
bLf	HSV-1 HSV-2	Vero cells	Immunofluorescence assay ELISA assay	Interference from differently metal-saturated Lf with viral binding to cell surface HS receptors. Possible antiviral activity through the interaction of Lf with the viral envelope	[100]
hLf/bLf	HSV-1 HSV-2	Human embryonic fibroblasts cells Murine L cells and derivatives (gro2C cells and sog9 cells)	Incubation of cells with Lf before viral adsorption and during adsorption and penetration steps Staining with crystal violet	Observed inhibition of HSV-1 infectivity dependent on Lf interaction with cell surface GAG chains of HS and CS Interference of Lf with the binding of viral gC to cell surface HS and/or CS	[101]
bLf	HSV-1	GMK cells	Time-of-addition assay SDS-PAGE, Western and far-Western blot Immunoprecipitation	Lf dose-dependent blocking of viral adsorption by competing for HS cell receptors Cell-to-cell viral spread inhibition caused by interaction of Lf with ICP-5 and VP-16 viral proteins	[102]
hLf/bLf	HSV-2	GMK cells	Staining with crystal violet	Possible blocking of non-GAG virus receptor by bLf Observed interaction between hLf and viral structures present in mutant virus HSV-2 gC-neg1	[103]
hLf	EBV	Primary B cells	Flow cytometry Q-PCR Coimmunoprecipitation SDS-PAGE and Western blot Tissue microarray and immunohistochemistry	Direct binding of Lf to CD21 cell receptors	[104]
hLf/bLf	Poliovirus	Vero cells	Indirect immunofluorescence	Inhibition of viral replication in presence of apo- and holo-Lf Antiviral effects detected with Zinc-saturated Lf when incubated with cells after the viral attachment	[105]
hLf/bLf	HCV	HepG2 cells	Far-Western blot Pull-down assay and immunoprecipitation	Binding of Lf to viral envelope proteins E1/E2	[106]
hLf/bLf	HCV	PH5CH8 cells	Preincubation of viral particles and cells with Lf Nested RT-PCR	Prevention of infection of cells in presence of virus preincubated with Lf	[107]
bLf	HCV	MT-2C T cells	Nested RT-PCR	Inhibition of viral entry to the cells by bLf interacting with HCV after mixing of bLF and HCV inoculum	[108]
rcLf/ncLf	HCV	Huh7.5 cells	Nested RT-PCR Western blot	Direct neutralization effects on the viral particle Prevention of viral entry into cells and inhibition of viral replication in infected cells	[109]
hLf	HCV	Huh-7 cells	Dose-response inhibition assay Immunofluorescence and SDS-PAGE	Inhibition of viral ATPase/Helicase NS3 protein mediated by direct interaction between Lf and allosteric binding site on NS3	[110]
bLf	HAdV	Human epidermoid carcinoma larynx (HEp-2) cells Viral particle	Preincubation of viral particles with Lf SDS-PAGE and Western blot Transmission electron microscopy	Binding of Lf to viral particle and external III and IIIa structural polypeptides	[111]
hLf/hLfcin	HAdV	A549 cells	Fiber knob binding experiments Surface plasmon resonance	Binding of Lfcin to hexon protein of viral capsid	[112]
hLf/bLf	CHV	MDCK cells	Preincubation of viral particles and cells with Lf SDS-PAGE and far-Western blot	Binding of bLf with CHV-binding protein in both apo- and holo-form Anti-viral activity of hLf observed	[113]
bLf	Echovirus	GMK cells	Preincubation of viral particles with Lf Immunofluorescence assay ELISA assay Transmission electron microscopy	Observed interaction of Lf with cell surface glycosaminoglycan chains and viral capsid proteins Prevention of viral genome delivery into cytoplasm	[114]
bLf	EV71	RD and SK-N-SH cells	ELISA assay and indirect fluorescent stain	Binding of Lf to VP1 protein of EV71 and target cells Induction of IFN-α expression in SK-N-SH cells and inhibition of EV71-induced IL-6 production	[115]
bLf	H1N1 Influenza A	MDCK cells	Time-of-addition assay ELISA assay Hemolysis inhibition assay	Lactoferrin binds to hemagglutinin and fusion peptide	[32]
hLf	SARS-CoV-2	RBD region/ACE2 receptor	2D Zernike polynomial expansion	Computational definition of regions of Lf with high affinity towards sialic acid and RBD	[116]
hLf	SARS-CoV-2	RBD region/ACE2 receptor	Biolayer interferometry Latex nanoparticle-enhanced turbidimetry	Binding of Lf with ACE2 and observed inhibiting effects in viral interaction with receptor	[117]

## References

[1] Lambert L.A. (2012). Molecular Evolution of the Transferrin Family and Associated Receptors. Biochim. Biophys. Acta (BBA)-Gen. Subj..

[2] Baker E.N., Anderson B.F., Baker H.M., Day C.L., Haridas M., Norris G.E., Rumball S.V., Smith C.A., Thomas D.H. (1994). Three-Dimensional Structure of Lactoferrin in Various Functional States. Adv. Exp. Med. Biol..

[3] Lambert L.A., Perri H., Halbrooks P.J., Mason A.B. (2005). Evolution of the Transferrin Family: Conservation of Residues Associated with Iron and Anion Binding. Comp. Biochem. Physiol. B Biochem. Mol. Biol..

[4] Park I., Schaeffer E., Sidoli A., Baralle F.E., Cohen G.N., Zakin M.M. (1985). Organization of the Human Transferrin Gene: Direct Evidence That It Originated by Gene Duplication. Proc. Natl. Acad. Sci. USA.

[5] Masson P.L., Heremans J.F., Dive C.H. (1966). An Iron-Binding Protein Common to Many External Secretions. Clin. Chim. Acta.

[6] Berlov M.N., Korableva E.S., Andreeva Y.V., Ovchinnikova T.V., Kokryakov V.N. (2007). Lactoferrin from Canine Neutrophils: Isolation and Physicochemical and Antimicrobial Properties. Biochemistry.

[7] Anderson B.F., Baker H.M., Dodson E.J., Norris G.E., Rumball S.V., Waters J.M., Baker E.N. (1987). Structure of Human Lactoferrin at 3.2 Å Resolution. Proc. Natl. Acad. Sci. USA.

[8] Anderson B.F., Baker H.M., Norris G.E., Rice D.W., Baker E.N. (1989). Structure of Human Lactoferrin: Crystallographic Structure Analysis and Refinement at 2.8 Å Resolution. J. Mol. Biol..

[9] Haridas M., Anderson B.F., Baker E.N. (1995). Structure of Human Diferric Lactoferrin Refined at 2.2 Å Resolution. Acta Crystallogr. D Biol. Crystallogr..

[10] Moore S.A., Anderson B.F., Groom C.R., Haridas M., Baker E.N. (1997). Three-Dimensional Structure of Diferric Bovine Lactoferrin at 2.8 Å Resolution. J. Mol. Biol..

[11] Khan J.A., Kumar P., Paramasivam M., Yadav R.S., Sahani M.S., Sharma S., Srinivasan A., Singh T.P. (2001). Camel Lactoferrin, a Transferrin-Cum-Lactoferrin: Crystal Structure of Camel Apolactoferrin at 2.6 Å Resolution and Structural Basis of Its Dual Role. J. Mol. Biol..

[12] Karthikeyan S., Yadav S., Paramasivam M., Srinivasan A., Singh T.P. (2000). Structure of Buffalo Lactoferrin at 3.3 Å Resolution at 277 K. Acta Crystallogr. D Biol. Crystallogr..

[13] Karthikeyan S., Paramasivam M., Yadav S., Srinivasan A., Singh T.P. (1999). Structure of Buffalo Lactoferrin at 2.5 Å Resolution Using Crystals Grown at 303 K Shows Different Orientations of the N and C Lobes. Acta Crystallogr. D Biol. Crystallogr..

[14] Sharma A.K., Paramasivam M., Srinivasan A., Yadav M.P., Singh T.P. (1999). Three-Dimensional Structure of Mare Diferric Lactoferrin at 2.6 Å Resolution. J. Mol. Biol..

[15] Baker H.M., Baker E.N. (2004). Lactoferrin and Iron: Structural and Dynamic Aspects of Binding and Release. BioMetals.

[16] Baker E.N., Baker H.M. (2005). Lactoferrin. Cell. Mol. Life Sci..

[17] Mason A.B., Halbrooks P.J., James N.G., Connolly S.A., Larouche J.R., Smith V.C., MacGillivray R.T.A., Chasteen N.D. (2005). Mutational Analysis of C-Lobe Ligands of Human Serum Transferrin: Insights into the Mechanism of Iron Release. Biochemistry.

[18] Zlatina K., Galuska S.P. (2021). The N-Glycans of Lactoferrin: More than Just a Sweet Decoration. Biochem. Cell Biol..

[19] Karav S., German J., Rouquié C., Le Parc A., Barile D. (2017). Studying Lactoferrin N-Glycosylation. Int. J. Mol. Sci..

[20] Van Veen H.A., Geerts M.E.J., Van Berkel P.H.C., Nuijens J.H. (2004). The Role of N-Linked Glycosylation in the Protection of Human and Bovine Lactoferrin against Tryptic Proteolysis. Eur. J. Biochem..

[21] van Berkel P.H.C., van Veen H.A., Geerts M.E.J., de Boer H.A., Nuijens J.H. (1996). Heterogeneity in Utilization of N-Glycosylation Sites Asn624 and Asn138 in Human Lactoferrin: A Study with Glycosylation-Site Mutants. Biochem. J..

[22] Wei Z., Nishimura T., Yoshida S. (2000). Presence of a Glycan at a Potential N-Glycosylation Site, Asn-281, of Bovine Lactoferrin. J. Dairy Sci..

[23] Ye X.-Y., Nishimura T., Yoshida S. (1997). Characterization of the Protein and Glycan Moieties in Different Forms of Bovine Lactoferrin. Biosci. Biotechnol. Biochem..

[24] Legrand D., Mazurier J., Colavizza D., Montreuil J., Spik G. (1990). Properties of the Iron-Binding Site of the N-Terminal Lobe of Human and Bovine Lactotransferrins. Importance of the Glycan Moiety and of the Non-Covalent Interactions between the N- and C-Terminal Lobes in the Stability of the Iron-Binding Site. Biochem. J..

[25] Li Z., Furmanski P. (1995). Role of Sialic Acid Residues in Iron Binding by Human Lactoferrin-α. Chin. J. Cancer Res..

[26] Barboza M., Pinzon J., Wickramasinghe S., Froehlich J.W., Moeller I., Smilowitz J.T., Ruhaak L.R., Huang J., Lönnerdal B., German J.B. (2012). Glycosylation of Human Milk Lactoferrin Exhibits Dynamic Changes During Early Lactation Enhancing Its Role in Pathogenic Bacteria-Host Interactions. Mol. Cell. Proteom..

[27] Rossi P., Giansanti F., Boffi A., Ajello M., Valenti P., Chiancone E., Antonini G. (2002). Ca^2+^ Binding to Bovine Lactoferrin Enhances Protein Stability and Influences the Release of Bacterial Lipopolysaccharide. Biochem. Cell Biol..

[28] Karabi Z., Moradian F., Kheirabadi M. (2022). The Effect of Lactoferrin on ULK1 and ATG13 Genes Expression in Breast Cancer Cell Line MCF7 and Bioinformatics Studies of Protein Interaction between Lactoferrin and the Autophagy Initiation Complex. Cell Biochem. Biophys..

[29] Cutone A., Rosa L., Ianiro G., Lepanto M.S., Bonaccorsi di Patti M.C., Valenti P., Musci G. (2020). Lactoferrin’s Anti-Cancer Properties: Safety, Selectivity, and Wide Range of Action. Biomolecules.

[30] Sharma S., Sinha M., Kaushik S., Kaur P., Singh T.P. (2013). C-Lobe of Lactoferrin: The Whole Story of the Half-Molecule. Biochem. Res. Int..

[31] Di Biase A.M., Pietrantoni A., Tinari A., Siciliano R., Valenti P., Antonini G., Seganti L., Superti F. (2003). Heparin-interacting Sites of Bovine Lactoferrin Are Involved in Anti-adenovirus Activity. J. Med. Virol..

[32] Superti F., Agamennone M., Pietrantoni A., Ammendolia M.G. (2019). Bovine Lactoferrin Prevents Influenza A Virus Infection by Interfering with the Fusogenic Function of Viral Hemagglutinin. Viruses.

[33] Kim W.-S., Shimazaki K., Tamura T. (2006). Expression of Bovine Lactoferrin C-Lobe in *Rhodococcus erythropolis* and Its Purification and Characterization. Biosci. Biotechnol. Biochem..

[34] Cao J. (2022). Functional Divergence of the N-Lobe and C-Lobe of Transferrin Gene in *Pungitius sinensis* (Amur Stickleback). Animals.

[35] Kim W.-S., Ohashi M., Shimazaki K. (2016). Inhibitory Effects of Synthetic Peptides Containing Bovine Lactoferrin C-Lobe Sequence on Bacterial Growth. Korean J. Food Sci. Anim. Resour..

[36] Jin L., Li L., Zhang W., Zhang R., Xu Y. (2022). Heterologous Expression of Bovine Lactoferrin C-Lobe in *Bacillus subtilis* and Comparison of Its Antibacterial Activity with N-Lobe. Syst. Microbiol. Biomanuf..

[37] D’Onofrio A., Crawford J.M., Stewart E.J., Witt K., Gavrish E., Epstein S., Clardy J., Lewis K. (2010). Siderophores from Neighboring Organisms Promote the Growth of Uncultured Bacteria. Chem. Biol..

[38] Morgenthau A., Livingstone M., Adamiak P., Schryvers A.B. (2012). The Role of Lactoferrin Binding Protein B in Mediating Protection against Human Lactoferricin 1. Biochem. Cell Biol..

[39] Ogunnariwo J.A., Schryvers A.B. (2001). Characterization of a Novel Transferrin Receptor in Bovine Strains of *Pasteurella multocida*. J. Bacteriol..

[40] Ekins A., Bahrami F., Sijercic A., Maret D., Niven D.F. (2004). *Haemophilus somnus* Possesses Two Systems for Acquisition of Transferrin-Bound Iron. J. Bacteriol..

[41] Barber M.F., Elde N.C. (2014). Escape from Bacterial Iron Piracy through Rapid Evolution of Transferrin. Science.

[42] Ogunnariwo J.A., Schryvers A.B. (1992). Correlation between the Ability of *Haemophilus paragallinarum* to Acquire Ovotransferrin-Bound Iron and the Expression of Ovotransferrin-Specific Receptors. Avian Dis..

[43] Schryvers A.B., Morris L.J. (1988). Identification and Characterization of the Human Lactoferrin-Binding Protein from *Neisseria meningitidis*. Infect. Immun..

[44] Schryvers A.B., Lee B.C. (1989). Comparative Analysis of the Transferrin and Lactoferrin Binding Proteins in the Family *Neisseriaceae*. Can. J. Microbiol..

[45] Bonnah R.A., Yu R., Schryvers A.B. (1995). Biochemical Analysis of Lactoferrin Receptors in the *Neisseriaceae*: Identification of a Second Bacterial Lactoferrin Receptor Protein. Microb. Pathog..

[46] Bonnah R.A., Schryvers A.B. (1998). Preparation and Characterization of *Neisseria meningitidis* Mutants Deficient in Production of the Human Lactoferrin-Binding Proteins LbpA and LbpB. J. Bacteriol..

[47] Yu R.-H., Schryvers A.B. (2002). Bacterial Lactoferrin Receptors: Insights from Characterizing the *Moraxella bovis* Receptors. Biochem. Cell Biol..

[48] Du R.-P., Wang Q., Yang Y.-P., Schryvers A.B., Chong P., Klein M.H., Loosmore S.M. (1998). Cloning and Expression of the *Moraxella catarrhalis* Lactoferrin Receptor Genes. Infect. Immun..

[49] Bonnah R.A., Wong H., Loosmore S.M., Schryvers A.B. (1999). Characterization of *Moraxella* (*Branhamella*) *catarrhalis* LbpB, LbpA, and Lactoferrin Receptor Orf3 Isogenic Mutants. Infect. Immun..

[50] Ostan N.K.H., Moraes T.F., Schryvers A.B. (2021). Lactoferrin Receptors in Gram-Negative Bacteria: An Evolutionary Perspective. Biochem. Cell Biol..

[51] Morgenthau A., Beddek A., Schryvers A.B. (2014). The Negatively Charged Regions of Lactoferrin Binding Protein B, an Adaptation against Anti-Microbial Peptides. PLoS ONE.

[52] Brooks C.L., Arutyunova E., Lemieux M.J. (2014). The Structure of Lactoferrin-Binding Protein B from *Neisseria meningitidis* Suggests Roles in Iron Acquisition and Neutralization of Host Defences. Acta Crystallogr. F Struct. Biol. Commun..

[53] Arutyunova E., Brooks C.L., Beddek A., Mak M.W., Schryvers A.B., Lemieux M.J. (2012). Crystal Structure of the N-Lobe of Lactoferrin Binding Protein B from *Moraxella bovis*. Biochem. Cell Biol..

[54] Arnold R.R., Cole M.F., McGhee J.R. (1977). A Bactericidal Effect for Human Lactoferrin. Science.

[55] Yamauchi K., Tomita M., Giehl T.J., Ellison R.T. (1993). Antibacterial Activity of Lactoferrin and a Pepsin-Derived Lactoferrin Peptide Fragment. Infect. Immun..

[56] Ellison R.T., Giehl T.J. (1991). Killing of Gram-Negative Bacteria by Lactoferrin and Lysozyme. J. Clin. Investig..

[57] Ellison R.T., Giehl T.J., LaForce F.M. (1988). Damage of the Outer Membrane of Enteric Gram-Negative Bacteria by Lactoferrin and Transferrin. Infect. Immun..

[58] Ellison R.T., LaForce F.M., Giehl T.J., Boose D.S., Dunn B.E. (1990). Lactoferrin and Transferrin Damage of the Gram-Negative Outer Membrane Is Modulated by Ca^2+^ and Mg^2+^. J. Gen. Microbiol..

[59] Elass-Rochard E., Roseanu A., Legrand D., Trif M., Salmon V., Motas C., Montreuil J., Spik G. (1995). Lactoferrin-Lipopolysaccharide Interaction: Involvement of the 28-34 Loop Region of Human Lactoferrin in the High-Affinity Binding to *Escherichia coli* 055B5 Lipopolysaccharide. Biochem. J..

[60] Nikaido H. (2003). Molecular Basis of Bacterial Outer Membrane Permeability Revisited. Microbiol. Mol. Biol. Rev..

[61] Appelmelk B.J., An Y.Q., Geerts M., Thijs B.G., De Boer H.A., MacLaren D.M., De Graaff J., Nuijens J.H. (1994). Lactoferrin Is a Lipid A-Binding Protein. Infect. Immun..

[62] Wang D., Pabst K.M., Aida Y., Pabst M.J. (1995). Lipopolysaccharide-Inactivating Activity of Neutrophils Is Due to Lactoferrin. J. Leukoc. Biol..

[63] Machnicki M., Zimecki M., Zagulski T. (1993). Lactoferrin Regulates the Release of Tumor Necrosis Factor Alpha and Interleukin 6 in Vivo. Int. J. Exp. Pathol..

[64] van Berkel P.H.C., Geerts E.J.M., van Veen A.H., Mericskay M., De Boer A.H., Nuijens H.J. (1997). N-Terminal Stretch Arg2, Arg3, Arg4 and Arg5 of Human Lactoferrin Is Essential for Binding to Heparin, Bacterial Lipopolysaccharide, Human Lysozyme and DNA. Biochem. J..

[65] Schryvers A.B., Morris L.J. (1988). Identification and Characterization of the Transferrin Receptor from *Neisseria meningitidis*. Mol. Microbiol..

[66] Mickelsen P.A., Blackman E., Sparling P.F. (1982). Ability of *Neisseria gonorrhoeae*, *Neisseria geningitidis*, and Commensal *Neisseria* Species to Obtain Iron from Lactoferrin. Infect. Immun..

[67] Gray-Owen S.D., Schyvers A.B. (1996). Bacterial Transferrin and Lactoferrin Receptors. Trends Microbiol..

[68] Morgenthau A., Pogoutse A., Adamiak P., Moraes T.F., Schryvers A.B. (2013). Bacterial Receptors for Host Transferrin and Lactoferrin: Molecular Mechanisms and Role in Host–Microbe Interactions. Future Microbiol..

[69] Moraes T.F., Yu R., Strynadka N.C.J., Schryvers A.B. (2009). Insights into the Bacterial Transferrin Receptor: The Structure of Transferrin-Binding Protein B from *Actinobacillus pleuropneumoniae*. Mol. Cell.

[70] Yang X., Yu R., Calmettes C., Moraes T.F., Schryvers A.B. (2011). Anchor Peptide of Transferrin-Binding Protein B Is Required for Interaction with Transferrin-Binding Protein A. J. Biol. Chem..

[71] Noinaj N., Easley N.C., Oke M., Mizuno N., Gumbart J., Boura E., Steere A.N., Zak O., Aisen P., Tajkhorshid E. (2012). Structural Basis for Iron Piracy by Pathogenic *Neisseria*. Nature.

[72] Noto J.M., Cornelissen C.N. (2008). Identification of TbpA Residues Required for Transferrin-Iron Utilization by *Neisseria gonorrhoeae*. Infect. Immun..

[73] Renauld-Mongénie G., Poncet D., Mignon M., Fraysse S., Chabanel C., Danve B., Krell T., Quentin-Millet M.-J. (2004). Role of Transferrin Receptor from a *Neisseria meningitidis* TbpB Isotype II Strain in Human Transferrin Binding and Virulence. Infect. Immun..

[74] Wong H., Schryvers A.B. (2003). Bacterial Lactoferrin-Binding Protein A Binds to Both Domains of the Human Lactoferrin C-Lobe. Microbiology.

[75] Noinaj N., Cornelissen C.N., Buchanan S.K. (2013). Structural Insight into the Lactoferrin Receptors from Pathogenic *Neisseria*. J. Struct. Biol..

[76] Biswas G.D., Anderson J.E., Chen C.-J., Cornelissen C.N., Sparling P.F. (1999). Identification and Functional Characterization of the *Neisseria gonorrhoeae* LbpB Gene Product. Infect. Immun..

[77] Biswas G.D., Sparling P.F. (1995). Characterization of LbpA, the Structural Gene for a Lactoferrin Receptor in *Neisseria gonorrhoeae*. Infect. Immun..

[78] Pettersson A., Maas A., Tommassen J. (1994). Identification of the IroA Gene Product of *Neisseria meningitidis* as a Lactoferrin Receptor. J. Bacteriol..

[79] Prinz T., Meyer M., Pettersson A., Tommassen J. (1999). Structural Characterization of the Lactoferrin Receptor from *Neisseria meningitidis*. J. Bacteriol..

[80] Yadav R., Govindan S., Daczkowski C., Mesecar A., Chakravarthy S., Noinaj N. (2021). Structural Insight into the Dual Function of LbpB in Mediating *Neisserial* Pathogenesis. Elife.

[81] Sill C., Biehl R., Hoffmann B., Radulescu A., Appavou M.-S., Farago B., Merkel R., Richter D. (2016). Structure and Domain Dynamics of Human Lactoferrin in Solution and the Influence of Fe(III)-Ion Ligand Binding. BMC Biophys..

[82] Naidu S.S., Svensson U., Kishore A.R., Naidu A.S. (1993). Relationship between Anti-bacterial Activity and Porin Binding of Lactoferrin in *Escherichia coli* and *Salmonella typhimurium*. Antimicrob. Agents Chemother..

[83] Erdei J., Forsgren A., Naidu A.S. (1994). Lactoferrin Binds to Porins OmpF and OmpC in *Escherichia coli*. Infect. Immun..

[84] Sallmann F.R., Baveye-Descamps S., Pattus F., Salmon V., Branza N., Spik G., Legrand D. (1999). Porins OmpC and PhoE of *Escherichia coli* as Specific Cell-Surface Targets of Human Lactoferrin. J. Biol. Chem..

[85] Ziere G.J., Bijsterbosch M.K., van Berkel T.J. (1993). Removal of 14 N-Terminal Amino Acids of Lactoferrin Enhances Its Affinity for Parenchymal Liver Cells and Potentiates the Inhibition of Beta- Very Low Density Lipoprotein Binding. J. Biol. Chem..

[86] Ziere G.J., Kruijt J.K., Bijsterbosch M.K., van Berkel T.J.C. (1996). Recognition of Lactoferrin and Aminopeptidase M-Modified Lactoferrin by the Liver: Involvement of Proteoglycans and the Remnant Receptor. Biochem. J..

[87] Legrand D., Mazurier J., Elass A., Rochard E., Vergoten G., Maes P., Montreuil J., Spik G. (1992). Molecular Interactions between Human Lactotransferrin and the Phytohemagglutinin-Activated Human Lymphocyte Lactotransferrin Receptor Lie in Two Loop-Containing Regions of the N-Terminal Domain I of Human Lactotransferrin. Biochemistry.

[88] Huettinger M., Retzek H., Hermann M., Goldenberg H. (1992). Lactoferrin Specifically Inhibits Endocytosis of Chylomicron Remnants but Not Alpha-Macroglobulin. J. Biol. Chem..

[89] Legrand D., Van Berkel P.H.C., Salmon V., Van Veen A.H., Slomianny M.-C., Nuijens H.J., Spik G. (1997). The N-Terminal Arg2, Arg3 and Arg4 of Human Lactoferrin Interact with Sulphated Molecules but Not with the Receptor Present on Jurkat Human Lymphoblastic T-Cells. Biochem. J..

[90] Mann D.M., Romm E., Migliorini M. (1994). Delineation of the Glycosaminoglycan-Binding Site in the Human Inflammatory Response Protein Lactoferrin. J. Biol. Chem..

[91] Shimazaki K., Tazume T., Uji K., Tanaka M., Kumura H., Mikawa K., Shimo-oka T. (1998). Properties of a Heparin-Binding Peptide Derived from Bovine Lactoferrin. J. Dairy Sci..

[92] Wu H.F., Monroe D.M., Church F.C. (1995). Characterization of the Glycosaminoglycan-Binding Region of Lactoferrin. Arch. Biochem. Biophys..

[93] Lévy-Toledano S., Grelac F., Caen J.P., Maclouf J. (1995). KRDS, a Peptide Derived from Human Lactotransferrin, Inhibits Thrombin-Induced Thromboxane Synthesis by a Cyclooxygenase-Independent Mechanism. Thromb. Haemost..

[94] Bellamy W., Takase M., Yamauchi K., Wakabayashi H., Kawase K., Tomita M. (1992). Identification of the Bactericidal Domain of Lactoferrin. Biochim. Biophys. Acta (BBA)-Protein Struct. Mol. Enzymol..

[95] Elass-Rochard E., Legrand D., Salmon V., Roseanu A., Trif M., Tobias P.S., Mazurier J., Spik G. (1998). Lactoferrin Inhibits the Endotoxin Interaction with CD14 by Competition with the Lipopolysaccharide-Binding Protein. Infect. Immun..

[96] Harmsen M.C., Swart P.J., Bethune M.-P.d., Pauwels R., Clercq E.D., The T.B., Meijer D.K.F. (1995). Antiviral Effects of Plasma and Milk Proteins: Lactoferrin Shows Potent Activity against Both Human Immunodeficiency Virus and Human Cytomegalovirus Replication In Vitro. J. Infect. Dis..

[97] Berkhout B., van Wamel J.L.B., Beljaars L., Meijer D.K.F., Visser S., Floris R. (2002). Characterization of the Anti-HIV Effects of Native Lactoferrin and Other Milk Proteins and Protein-Derived Peptides. Antivir. Res..

[98] Groot F., Geijtenbeek T.B.H., Sanders R.W., Baldwin C.E., Sanchez-Hernandez M., Floris R., van Kooyk Y., de Jong E.C., Berkhout B. (2005). Lactoferrin Prevents Dendritic Cell-Mediated Human Immunodeficiency Virus Type 1 Transmission by Blocking the DC-SIGN--Gp120 Interaction. J. Virol..

[99] Marchetti M., Longhi C., Conte M.P., Pisani S., Valenti P., Seganti L. (1996). Lactoferrin Inhibits Herpes Simplex Virus Type 1 Adsorption to Vero Cells. Antivir. Res..

[100] Marchetti M., Pisani S., Antonini G., Valenti P., Seganti L., Orsi N. (1998). Metal Complexes of Bovine Lactoferrin Inhibit in Vitro Replication of Herpes Simplex Virus Type 1 and 2. Biometals.

[101] Marchetti M., Trybala E., Superti F., Johansson M., Bergström T. (2004). Inhibition of Herpes Simplex Virus Infection by Lactoferrin Is Dependent on Interference with the Virus Binding to Glycosaminoglycans. Virology.

[102] Ammendolia M.G., Marchetti M., Superti F. (2007). Bovine Lactoferrin Prevents the Entry and Intercellular Spread of Herpes Simplex Virus Type 1 in Green Monkey Kidney Cells. Antivir. Res..

[103] Marchetti M., Ammendolia M.G., Superti F. (2009). Glycosaminoglycans Are Not Indispensable for the Anti-Herpes Simplex Virus Type 2 Activity of Lactoferrin. Biochimie.

[104] Zheng Y., Zhang W., Ye Q., Zhou Y., Xiong W., He W., Deng M., Zhou M., Guo X., Chen P. (2012). Inhibition of Epstein-Barr Virus Infection by Lactoferrin. J. Innate Immun..

[105] Marchetti M., Superti F., Ammendolia M.G., Rossi P., Valenti P., Seganti L. (1999). Inhibition of Poliovirus Type 1 Infection by Iron-, Manganese- and Zinc-Saturated Lactoferrin. Med. Microbiol. Immunol..

[106] Yi M., Kaneko S., Yu D., Murakami S. (1997). Hepatitis C Virus Envelope Proteins Bind Lactoferrin. J. Virol..

[107] Ikeda M., Sugiyama K., Tanaka T., Tanaka K., Sekihara H., Shimotohno K., Kato N. (1998). Lactoferrin Markedly Inhibits Hepatitis C Virus Infection in Cultured Human Hepatocytes. Biochem. Biophys. Res. Commun..

[108] Ikeda M., Nozaki A., Sugiyama K., Tanaka T., Naganuma A., Tanaka K., Sekihara H., Shimotohno K., Saito M., Kato N. (2000). Characterization of Antiviral Activity of Lactoferrin against Hepatitis C Virus Infection in Human Cultured Cells. Virus Res..

[109] Liao Y., El-Fakkarany E., Lönnerdal B., Redwan E.M. (2012). Inhibitory Effects of Native and Recombinant Full-Length Camel Lactoferrin and Its N and C Lobes on Hepatitis C Virus Infection of Huh7.5 Cells. J. Med. Microbiol..

[110] Picard-Jean F., Bouchard S., Larivée G., Bisaillon M. (2014). The Intracellular Inhibition of HCV Replication Represents a Novel Mechanism of Action by the Innate Immune Lactoferrin Protein. Antivir. Res..

[111] Pietrantoni A., Di Biase A.M., Tinari A., Marchetti M., Valenti P., Seganti L., Superti F. (2003). Bovine Lactoferrin Inhibits Adenovirus Infection by Interacting with Viral Structural Polypeptides. Antimicrob. Agents Chemother..

[112] Persson B.D., Lenman A., Frängsmyr L., Schmid M., Ahlm C., Plückthun A., Jenssen H., Arnberg N. (2020). Lactoferrin-Hexon Interactions Mediate CAR-Independent Adenovirus Infection of Human Respiratory Cells. J. Virol..

[113] Tanaka T., Nakatani S., Xuan X., Kumura H., Igarashi I., Shimazaki K. (2003). Antiviral Activity of Lactoferrin against Canine Herpesvirus. Antivir. Res..

[114] Ammendolia M.G., Pietrantoni A., Tinari A., Valenti P., Superti F. (2007). Bovine Lactoferrin Inhibits Echovirus Endocytic Pathway by Interacting with Viral Structural Polypeptides. Antivir. Res..

[115] Weng T., Chen L., Shyu H., Chen S., Wang J., Yu C., Lei H., Yeh T. (2005). Lactoferrin Inhibits Enterovirus 71 Infection by Binding to VP1 Protein and Host Cells. Antivir. Res..

[116] Miotto M., Di Rienzo L., Bò L., Boffi A., Ruocco G., Milanetti E. (2021). Molecular Mechanisms Behind Anti SARS-CoV-2 Action of Lactoferrin. Front. Mol. Biosci..

[117] Piacentini R., Centi L., Miotto M., Milanetti E., Di Rienzo L., Pitea M., Piazza P., Ruocco G., Boffi A., Parisi G. (2022). Lactoferrin Inhibition of the Complex Formation between ACE2 Receptor and SARS-CoV-2 Recognition Binding Domain. Int. J. Mol. Sci..

[118] Tu M., Xu S., Xu Z., Cheng S., Wu D., Liu H., Du M. (2021). Identification of Dual-Function Bovine Lactoferrin Peptides Released Using Simulated Gastrointestinal Digestion. Food Biosci..

[119] Berlutti F., Pantanella F., Natalizi T., Frioni A., Paesano R., Polimeni A., Valenti P. (2011). Antiviral Properties of Lactoferrin—A Natural Immunity Molecule. Molecules.

[120] Chang R., Ng T.B., Sun W.-Z. (2020). Lactoferrin as Potential Preventative and Adjunct Treatment for COVID-19. Int. J. Antimicrob. Agents.

[121] Zhao W., Li X., Yu Z., Wu S., Ding L., Liu J. (2022). Identification of Lactoferrin-Derived Peptides as Potential Inhibitors against the Main Protease of SARS-CoV-2. LWT.

[122] Cutone A., Rosa L., Bonaccorsi di Patti M.C., Iacovelli F., Conte M.P., Ianiro G., Romeo A., Campione E., Bianchi L., Valenti P. (2022). Lactoferrin Binding to SARS-CoV-2 Spike Glycoprotein Blocks Pseudoviral Entry and Relieves Iron Protein Dysregulation in Several In Vitro Models. Pharmaceutics.

[123] Kozakov D., Hall D.R., Xia B., Porter K.A., Padhorny D., Yueh C., Beglov D., Vajda S. (2017). The ClusPro Web Server for Protein–Protein Docking. Nat. Protoc..

[124] Campione E., Lanna C., Cosio T., Rosa L., Conte M.P., Iacovelli F., Romeo A., Falconi M., Del Vecchio C., Franchin E. (2021). Lactoferrin Against SARS-CoV-2: In Vitro and In Silico Evidences. Front. Pharmacol..

[125] Milanetti E., Miotto M., Di Rienzo L., Monti M., Gosti G., Ruocco G. (2021). 2D Zernike Polynomial Expansion: Finding the Protein-Protein Binding Regions. Comput. Struct. Biotechnol. J..

[126] Milanetti E., Miotto M., Di Rienzo L., Nagaraj M., Monti M., Golbek T.W., Go-sti G., Roeters S.J., Weidner T., Otzen D.E. (2021). In-Silico Evidence for a Two Receptor Based Strategy of SARS-CoV-2. Front. Mol. Biosci..

[127] Bò L., Miotto M., Di Rienzo L., Milanetti E., Ruocco G. (2021). Exploring the Association Between Sialic Acid and SARS-CoV-2 Spike Protein through a Molecular Dynamics-Based Approach. Front. Med. Technol..

[128] Monti M., Milanetti E., Frans M.T., Miotto M., Di Rienzo L., Baranov M.V., Gosti G., Somavarapu A.K., Nagaraj M., Golbek T.W. (2024). Two Receptor Binding Strategy of SARS-CoV-2 Is Mediated by Both the N-Terminal and Receptor-Binding Spike Domain. J. Phys. Chem. B.

[129] Nguyen L., McCord K.A., Bui D.T., Bouwman K.M., Kitova E.N., Elaish M., Kumawat D., Daskhan G.C., Tomris I., Han L. (2022). Sialic Acid-Containing Glycolipids Mediate Binding and Viral Entry of SARS-CoV-2. Nat. Chem. Biol..

[130] Unione L., Moure M.J., Lenza M.P., Oyenarte I., Ereño-Orbea J., Ardá A., Jiménez-Barbero J. (2022). The SARS-CoV-2 Spike Glycoprotein Directly Binds Exogeneous Sialic Acids: A NMR View. Angew. Chem. Int. Ed..

[131] Iacovelli F., Costanza G., Romeo A., Cosio T., Lanna C., Bagnulo A., Di Maio U., Sbardella A., Gaziano R., Grelli S. (2022). Interaction of Pelargonium Sidoides Compounds with Lactoferrin and SARS-CoV-2: Insights from Molecular Simulations. Int. J. Environ. Res. Public Health.

[132] Furlund C.B., Ulleberg E.K., Devold T.G., Flengsrud R., Jacobsen M., Sekse C., Holm H., Vegarud G.E. (2013). Identification of Lactoferrin Peptides Generated by Digestion with Human Gastrointestinal Enzymes. J. Dairy Sci..

[133] Kuwata H., Yip T.-T., Tomita M., Hutchens T.W. (1998). Direct Evidence of the Generation in Human Stomach of an Antimicrobial Peptide Domain (Lactoferricin) from Ingested Lactoferrin. Biochim. Biophys. Acta (BBA)-Protein Struct. Mol. Enzymol..

[134] Kuwata H., Yamauchi K., Teraguchi S., Hayasawa H., Ushida Y., Shimokawa Y., Toida T. (2001). Functional Fragments of Ingested Lactoferrin Are Resistant to Proteolytic Degradation in the Gastrointestinal Tract of Adult Rats. J. Nutr..

[135] Tomita M., Bellamy W., Takase M., Yamauchi K., Wakabayashi H., Kawase K. (1991). Potent Antibacterial Peptides Generated by Pepsin Digestion of Bovine Lactoferrin. J. Dairy Sci..

[136] Umeyama M., Kira A., Nishimura K., Naito A. (2006). Interactions of Bovine Lactoferricin with Acidic Phospholipid Bilayers and Its Antimicrobial Activity as Studied by Solid-State NMR. Biochim. Biophys. Acta (BBA)-Biomembr..

[137] Diarra M.S. (2003). Ultrastructural and Cytochemical Study of Cell Wall Modification by Lactoferrin, Lactoferricin and Penicillin G against *Staphylococcus aureus*. J. Electron. Microsc..

[138] Farnaud S., Spiller C., Moriarty L.C., Patel A., Gant V., Odell E.W., Evans R.W. (2004). Interactions of Lactoferricin-Derived Peptides with LPS and Anti-microbial Activity. FEMS Microbiol. Lett..

[139] Hunter H.N., Demcoe A.R., Jenssen H., Gutteberg T.J., Vogel H.J. (2005). Human Lactoferricin Is Partially Folded in Aqueous Solution and Is Better Stabilized in a Membrane Mimetic Solvent. Antimicrob. Agents Chemother..

[140] Gruden Š., Poklar Ulrih N. (2021). Diverse Mechanisms of Antimicrobial Activities of Lactoferrins, Lactoferricins, and Other Lactoferrin-Derived Peptides. Int. J. Mol. Sci..

[141] Chapple D.S., Hussain R., Joannou C.L., Hancock R.E.W., Odell E., Evans R.W., Siligardi G. (2004). Structure and Association of Human Lactoferrin Peptides with *Escherichia coli* Lipopolysaccharide. Antimicrob. Agents Chemother..

[142] Farnaud S., Patel A., Odell E.W., Evans R.W. (2004). Variation in Antimicrobial Activity of Lactoferricin-Derived Peptides Explained by Structure Modelling. FEMS Micro-Biol. Lett..

[143] Pei J., Xiong L., Bao P., Chu M., Yan P., Guo X. (2021). Secondary Structural Transformation of Bovine Lactoferricin Affects Its Antibacterial Activity. Probiotics Antimicrob. Proteins.

[144] Hilde Ulvatne L.H.V. (2001). Bactericidal Kinetics of 3 Lactoferricins Against *Staphylococcus aureus* and *Escherichia coli*. Scand. J. Infect. Dis..

[145] Liu Y., Han F., Xie Y., Wang Y. (2011). Comparative Antimicrobial Activity and Mechanism of Action of Bovine Lactoferricin-Derived Synthetic Peptides. BioMetals.

[146] Haug B.E., Svendsen J.S. (2001). The Role of Tryptophan in the Antibacterial Activity of a 15-residue Bovine Lactoferricin Peptide. J. Pept. Sci..

[147] Vorland L.H., Ulvatne H., Andersen J., Haukland H.H., Rekdal O., Svendsen J.S., Gutteberg T.J. (1999). Antibacterial Effects of Lactoferricin B. Scand. J. Infect. Dis..

[148] van der Kraan M.I.A., Groenink J., Nazmi K., Veerman E.C.I., Bolscher J.G.M., Nieuw Amerongen A. (2004). V Lactoferrampin: A Novel Antimicrobial Peptide in the N1-Domain of Bovine Lactoferrin. Peptides.

[149] Bolscher J.G.M., van der Kraan M.I.A., Nazmi K., Kalay H., Grün C.H., van’t Hof W., Veerman E.C.I., Nieuw Amerongen A.V. (2006). A One-Enzyme Strategy to Release an Antimicrobial Peptide from the LFampin-Domain of Bovine Lactoferrin. Peptides.

[150] Haney E., Nazmi K., Lau F., Bolscher J.G.M., Vogel H. (2009). Novel Lactoferrampin Antimicrobial Peptides Derived from Human Lactoferrin. Biochimie.

[151] Dijkshoorn L., Brouwer C.P.J.M., Bogaards S.J.P., Nemec A., van den Broek P.J., Nibbering P.H. (2004). The Synthetic N-Terminal Peptide of Human Lactoferrin, HLF(1-11), Is Highly Effective against Experimental Infection Caused by Multi-drug-Resistant *Acinetobacter baumannii*. Antimicrob. Agents Chemother..

[152] Faber C., Stallmann H.P., Lyaruu D.M., Joosten U., von Eiff C., van Nieuw Amerongen A., Wuisman P.I.J.M. (2005). Comparable Efficacies of the Antimicrobial Peptide Human Lactoferrin 1-11 and Gentamicin in a Chronic Methicillin-Resistant *Staphylococcus aureus* Osteomyelitis Model. Antimicrob. Agents Chemother..

[153] Denardi L.B., de Arruda Trindade P., Weiblen C., Ianiski L.B., Stibbe P.C., Pin-to S.C., Santurio J.M. (2022). In Vitro Activity of the Antimicrobial Peptides H-Lf1-11, MSI-78, LL-37, Fengycin 2B, and Magainin-2 against Clinically Important Bacteria. Braz. J. Microbiol..

[154] Lupetti A., Brouwer C.P.J.M., Bogaards S.J.P., Welling M.M., de Heer E., Campa M., van Dissel J.T., Friesen R.H.E., Nibbering P.H. (2007). Human Lactoferrin-Derived Peptide’s Antifungal Activities against Disseminated *Candida albicans* Infection. J. Infect. Dis..

[155] Lupetti A., Paulusma-Annema A., Welling M.M., Dogterom-Ballering H., Brouwer C.P.J.M., Senesi S., van Dissel J.T., Nibbering P.H. (2003). Synergistic Activity of the N-Terminal Peptide of Human Lactoferrin and Fluconazole against *Candida* Species. Antimicrob. Agents Chemother..

[156] Lupetti A., Paulusma-Annema A., Welling M.M., Senesi S., van Dissel J.T., Nibbering P.H. (2000). Candidacidal Activities of Human Lactoferrin Peptides Derived from the N Terminus. Antimicrob. Agents Chemother..

[157] Nibbering P.H., Ravensbergen E., Welling M.M., van Berkel L.A., van Berkel P.H., Pauwels E.K., Nuijens J.H. (2001). Human Lactoferrin and Peptides Derived from Its N Terminus Are Highly Effective against Infections with Antibiotic-Resistant Bacteria. Infect. Immun..

[158] Stallmann H.P. (2004). Osteomyelitis Prevention in Rabbits Using Antimicrobial Peptide HLF1-11- or Gentamicin-Containing Calcium Phosphate Cement. J. Anti-Microb. Chemother..

[159] van der Does A.M., Bogaards S.J.P., Ravensbergen B., Beekhuizen H., van Dissel J.T., Nibbering P.H. (2010). Antimicrobial Peptide HLF1-11 Directs Granulocyte-Macrophage Colony-Stimulating Factor-Driven Monocyte Differentiation toward Macrophages with Enhanced Recognition and Clearance of Pathogens. Antimicrob. Agents Chemother..

[160] van der Does A.M., Hensbergen P.J., Bogaards S.J., Cansoy M., Deelder A.M., van Leeuwen H.C., Drijfhout J.W., van Dissel J.T., Nibbering P.H. (2012). The Human Lactoferrin-Derived Peptide HLF1-11 Exerts Immunomodulatory Effects by Specific Inhibition of Myeloperoxidase Activity. J. Immunol..

[161] Sinha M., Kaushik S., Kaur P., Sharma S., Singh T.P. (2013). Antimicrobial Lactoferrin Peptides: The Hidden Players in the Protective Function of a Multifunctional Protein. Int. J. Pept..

[162] Dionysius D.A., Milne J.M. (1997). Antibacterial Peptides of Bovine Lactoferrin: Purification and Characterization. J. Dairy Sci..

[163] Recio I., Visser S. (1999). Two Ion-Exchange Chromatographic Methods for the Isolation of Antibacterial Peptides from Lactoferrin. J. Chromatogr. A.

[164] Kim W.-S., Kim P.-H., Shimazaki K.-I. (2016). Sensitivity of *Pseudomonas syringae* to Bovine Lactoferrin Hydrolysates and Identification of a Novel Inhibitory Peptide. Korean J. Food Sci. Anim. Resour..

[165] Lizzi A.R., Carnicelli V., Clarkson M.M., Nazzicone C., Segatore B., Celenza G., Aschi M., Dolo V., Strom R., Amicosante G. (2016). Bovine Lactoferrin and Its Tryptic Peptides: Antibacterial Activity against Different Species. Appl. Biochem. Microbiol..

[166] Rastogi N., Nagpal N., Alam H., Pandey S., Gautam L., Sinha M., Shin K., Manzoor N., Virdi J.S., Kaur P. (2014). Preparation and Antimicrobial Action of Three Tryptic Digested Functional Molecules of Bovine Lactoferrin. PLoS ONE.

[167] Hoek K.S., Milne J.M., Grieve P.A., Dionysius D.A., Smith R. (1997). Antibacterial Activity in Bovine Lactoferrin-Derived Peptides. Antimicrob. Agents Chemother..

[168] León-Sicairos N., Canizalez Roman A., De La Garza M., Reyes Lopez M., Zazueta-Beltran J., Nazmi K., Gomez-Gil B., Bolscher J.G.M. (2009). Bactericidal Effect of Lactoferrin and Lactoferrin Chimera against Halophilic *Vibrio parahaemolyticus*. Biochimie.

[169] León-Sicairos N., Angulo-Zamudio U.A., Vidal J.E., López-Torres C.A., Bolscher J.G.M., Nazmi K., Reyes-Cortes R., Reyes-López M., de la Garza M., Canizalez-Román A. (2014). Bactericidal Effect of Bovine Lactoferrin and Synthetic Peptide Lactoferrin Chimera in *Streptococcus pneumoniae* and the Decrease in LuxS Gene Expression by Lactoferrin. BioMetals.

[170] Sijbrandij T., Ligtenberg A.J., Nazmi K., van den Keijbus P.A.M., Veerman E.C.I., Bolscher J.G.M., Bikker F.J. (2018). LFchimera protects HeLa cells from invasion by *Yersinia* spp. in vitro. BioMetals.

[171] Puknun A., Bolscher J.G.M., Nazmi K., Veerman E.C.I., Tungpradabkul S., Wongratanacheewin S., Kanthawong S., Taweechaisupapong S. (2013). A Heterodimer Comprised of Two Bovine Lactoferrin Antimicrobial Peptides Exhibits Powerful Bactericidal Activity against *Burkholderia pseudomallei*. World J. Microbiol. Biotechnol..

[172] Haney E.F., Nazmi K., Bolscher J.G.M., Vogel H.J. (2012). Structural and Biophysical Characterization of an Antimicrobial Peptide Chimera Comprised of Lactoferricin and Lactoferrampin. Biochim. Biophys. Acta (BBA)-Biomembr..

[173] Bolscher J.G.M., Adao R., Nazmi K., Vandenkeybus P., Vanthof W., Nieuwamerongen A., Bastos M., Veerman E. (2009). Bactericidal Activity of LFchimera Is Stronger and Less Sensitive to Ionic Strength than Its Constituent Lactoferricin and Lactoferrampin Peptides. Biochimie.

[174] Sokolov A.V., Zakahrova E.T., Kostevich V.A., Samygina V.R., Vasilyev V.B. (2014). Lactoferrin, Myeloperoxidase, and Ceruloplasmin: Complementary Gearwheels Cranking Physiological and Pathological Processes. BioMetals.

[175] Sokolov A.V., Pulina M.O., Ageeva K.V., Ayrapetov M.I., Berlov M.N., Volgin G.N., Markov A.G., Yablonsky P.K., Kolodkin N.I., Zakharova E.T. (2007). Interaction of Ceruloplasmin, Lactoferrin, and Myeloperoxidase. Biochemistry.

[176] Sokolov A.V., Ageeva K.V., Pulina M.O., Zakharova E.T., Vasilyev V.B. (2009). Effect of Lactoferrin on Oxidative Features of Ceruloplasmin. BioMetals.

[177] Zakharova E.T., Shavlovski M.M., Bass M.G., Gridasova A.A., Pulina M.O., De Filippis V., Beltramini M., Di Muro P., Salvato B., Fontana A. (2000). Interaction of Lactoferrin with Ceruloplasmin. Arch. Biochem. Biophys..

[178] Pulina M.O., Zakharova E.T., Sokolov A.V., Shavlovski M.M., Bass M.G., Solovyov K.V., Kokryakov V.N., Vasilyev V.B. (2002). Studies of the Ceruloplasmin-Lactoferrin Complex. Biochem. Cell Biol..

[179] Barinov N.A., Vlasova I.I., Sokolov A.V., Kostevich V.A., Dubrovin E.V., Klinov D.V. (2018). High-Resolution Atomic Force Microscopy Visualization of Metallo-proteins and Their Complexes. Biochim. Biophys. Acta (BBA)-Gen. Subj..

[180] Sabatucci A., Vachette P., Vasilyev V.B., Beltramini M., Sokolov A., Pulina M., Salvato B., Angelucci C.B., Maccarrone M., Cozzani I. (2007). Structural Characterization of the Ceruloplasmin: Lactoferrin Complex in Solution. J. Mol. Biol..

[181] Sokolov A.V., Pulina M.O., Zakharova E.T., Susorova A.S., Runova O.L., Ko-lodkin N.I., Vasilyev V.B. (2006). Identification and Isolation from Breast Milk of Ceruloplasmin-Lactoferrin Complex. Biochemistry.

[182] Sokolov A.V., Voynova I.V., Kostevich V.A., Vlasenko A.Y., Zakharova E.T., Vasilyev V.B. (2017). Comparison of Interaction between Ceruloplasmin and Lactoferrin/Transferrin: To Bind or Not to Bind. Biochemistry.

[183] Sakajiri T., Nakatsuji M., Teraoka Y., Furuta K., Ikuta K., Shibusa K., Sugano E., Tomita H., Inui T., Yamamura T. (2021). Zinc Mediates the Interaction between Ceruloplasmin and Apo-Transferrin for the Efficient Transfer of Fe(III) Ions. Metallomics.

[184] Suzuki Y.A., Shin K., Lönnerdal B. (2001). Molecular Cloning and Functional Expression of a Human Intestinal Lactoferrin Receptor. Biochemistry.

[185] Suzuki Y.A., Wong H., Ashida K.-Y., Schryvers A.B., Lönnerdal B. (2008). The N1 Domain of Human Lactoferrin Is Required for Internalization by Caco-2 Cells and Targeting to the Nucleus. Biochemistry.

[186] Mancinelli R., Olivero F., Carpino G., Overi D., Rosa L., Lepanto M.S., Cutone A., Franchitto A., Alpini G., Onori P. (2018). Role of Lactoferrin and Its Receptors on Biliary Epithelium. BioMetals.

[187] Wesener D.A., Wangkanont K., McBride R., Song X., Kraft M.B., Hodges H.L., Zarling L.C., Splain R.A., Smith D.F., Cummings R.D. (2015). Recognition of Microbial Glycans by Human Intelectin-1. Nat. Struct. Mol. Biol..

[188] Takayama Y., Aoki R., Uchida R., Tajima A., Aoki-Yoshida A. (2017). Role of CXC Chemokine Receptor Type 4 as a Lactoferrin Receptor. Biochem. Cell Biol..

